# Single‐cell RNA Sequencing Identified Novel Nr4a1^+^ Ear2^+^ Anti‐Inflammatory Macrophage Phenotype under Myeloid‐TLR4 Dependent Regulation in Anti‐Glomerular Basement Membrane (GBM) Crescentic Glomerulonephritis (cGN)

**DOI:** 10.1002/advs.202200668

**Published:** 2022-04-28

**Authors:** Jiaoyi Chen, Xiao Ru Huang, Fuye Yang, Wai Han Yiu, Xueqing Yu, Sydney C. W. Tang, Hui Yao Lan

**Affiliations:** ^1^ Department of Medicine and Therapeutics Li Ka Shing Institute of Health Sciences and Lui Che Woo Institute of Innovative Medicine The Chinese University of Hong Kong Hong Kong 999077 P. R. China; ^2^ Guangdong‐Hong Kong Joint Laboratory on Immunological and Genetic Kidney Diseases Guangdong Academy of Medical Sciences Guangdong Provincial People's Hospital Guangzhou 510080 P. R. China; ^3^ Department of Nephrology The Second Affiliated Hospital of Zhejiang University School of Medicine Hangzhou Zhejiang 31009 P. R. China; ^4^ Division of Nephrology Department of Medicine The University of Hong Kong Hong Kong 999077 P. R. China; ^5^ The Chinese University of Hong Kong‐Guangdong Academy of Sciences/Guangdong Provincial People's Hospital Joint Research Laboratory on Immunological and Genetic Kidney Diseases The Chinese University of Hong Kong Hong Kong 999077 P. R. China

**Keywords:** anti‐GBM crescentic glomerulonephritis, macrophages, myeloid‐TLR4, Nr4a1/Ear2

## Abstract

Previously, this study demonstrates the critical role of myeloid specific TLR4 in macrophage‐mediated progressive renal injury in anti‐glomerular basement membrane (anti‐GBM) crescentic glomerulonephritis (cGN); however, the underlying mechanism remains largely unknown. In this study, single‐cell RNA sequencing (scRNA‐seq), pseudotime trajectories reconstruction, and motif enrichment analysis are used, and macrophage diversity in anti‐GBM cGN under tight regulation of myeloid‐TLR4 is uncovered. Most significantly, a myeloid‐TLR4 deletion‐induced novel reparative macrophage phenotype (*Nr4a1^+^* *Ear2+*) with significant upregulated anti‐inflammatory and tissue repair‐related signaling is discovered, thereby suppressing the M1 proinflammatory responses in anti‐GBM cGN. This is further demonstrated in vitro that deletion of TLR4 from bone marrow‐derived macrophages (BMDMs) induces the *Nr4a1/Ear2*‐expressing anti‐inflammatory macrophages while blocking LPS‐stimulated M1 proinflammatory responses. Mechanistically, activation of the Nr4a1/Ear2‐axis is recognized as a key mechanism through which deletion of myeloid‐TLR4 promotes the anti‐inflammatory macrophage differentiation in vivo and in vitro. This is confirmed by specifically silencing macrophage *Nr4a1* or *Ear2* to reverse the anti‐inflammatory effects on TLR4 deficient BMDMs upon LPS stimulation. In conclusion, the findings decode a previously unidentified role for a myeloid‐TLR4 dependent Nr4a1/Ear2 negative feedback mechanism in macrophage‐mediated progressive renal injury, implying that activation of Nr4a1‐Ear2 axis can be a novel and effective immunotherapy for anti‐GBM cGN.

## Graphical abstract

In this study, the authors discover that deletion of myeloid TLR4 inhibits immunologically mediated crescentic glomerulonephritis (cGN) by inducing a novel Nr4a1/Ear2‐expressing anti‐inflammatory macrophages while suppressing M1 proinflammatory responses. Findings from this study decode a previously unidentified role for a myeloid‐TLR4 dependent Nr4a1/Ear2 negative feedback mechanism in macrophage‐mediated progressive renal injury.

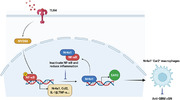

## 1 Introduction

It is widely reported that TLR4 and its downstream signaling play a critical role in the pathogenesis of autoimmune glomerulonephritis.^[^ ^1^, ^2^, ^3^ ^]^ Conventional TLR4 deletion has been reported to notably protect against renal dysfunction in experimental anti‐glomerular basement membrane (anti‐GBM) crescentic glomerulonephritis (cGN).^[^ ^3^ ^]^ Indeed, TLR4 contributes to the early and transient glomerular neutrophil influx in the first 24 h in nephrotoxic antibody‐induced anti‐GBM GN.^[^ ^1^ ^]^ However, the relative role of cell‐type specific TLR4, especially macrophage‐specific TLR4, remains largely unknown.

Increasing evidence has shown that macrophages play a critical role in the pathogenesis of kidney diseases,^[^ ^4^, ^5^ ^]^ supported by many studies in which macrophage deletion or inactivation can protect against anti‐GBM cGN.^[^ ^6^, ^7^, ^8^, ^9^ ^]^ Among macrophages, it has been well established that the M1 pro‐inflammatory macrophages are pathogenic while the M2 phenotype is renal protective in a number of kidney diseases including the anti‐GBM cGN.^[^ ^4^, ^5^, ^10^ ^]^ However, the role and mechanism of TLR4 in macrophage‐mediated anti‐GBM cGN remain largely unclear. To address this question, we established the myeloid cell‐specific TLR4 conditional knockout mice (*Tlr4^fl/fl^/LysM‐cre*) for experimental anti‐GBM cGN model. Our previous study demonstrated that deletion of myeloid‐TLR4 markedly alleviated progressive cGN and largely improved renal function by suppressing macrophage and neutrophil infiltration, and promoting macrophage polarization into the anti‐inflammatory M2 phenotype.^[^ ^11^ ^]^ However, transcriptional regulation through which deletion of myeloid TLR4 leads to the shift of macrophages from the M1 to M2 phenotype in anti‐GBM cGN remains to be elucidated.

With a view to intensively clarify the myeloid‐TLR4 dependent regulation on the comprehensive immune landscape in experimental anti‐GBM cGN, we used single‐cell RNA sequencing (scRNA‐seq) to obtain a novel and global insight into the complex immune landscape within the diseased kidney tissue. Additional combination of scRNA‐seq, pseudotime cell developmental trajectory reconstruction, and transcription factor (TF)‐centered motif enrichment analysis was further conducted to profile, for the first time to our knowledge, renal CD45^+^ leukocytes transcriptome at single‐cell resolution in experimental anti‐GBM cGN. More importantly, we also uncovered the mechanisms through which TLR4 regulates macrophage/monocyte differentiation and polarization via macrophage‐specific TFs and TF‐centric regulator networks enrichment in the present study. We found that deletion of myeloid‐specific TLR4 led to a remarkable transcriptome swift among monocyte/macrophage population from pro‐inflammatory toward the anti‐inflammatory phenotypes and thus suppressed macrophage immune response while promoting the renal repair process in anti‐GBM cGN. Surprisingly, we discovered a novel anti‐inflammatory macrophage subtype (*Nr4a1^+^ Ear2^+^*) uniquely derived from macrophages with TLR4‐deficiency in anti‐GBM cGN kidney, demonstrating a novel role for TLR4/Nr4a1/Ear2 feedback axis in regulating macrophage‐mediated renal injury, which was further explored in this study.

## 2 Results

### 2.1 Single‐Cell RNA Sequencing Uncovered the Diverse Immune Landscape in Anti‐GBM cGN

**Figure 1:**
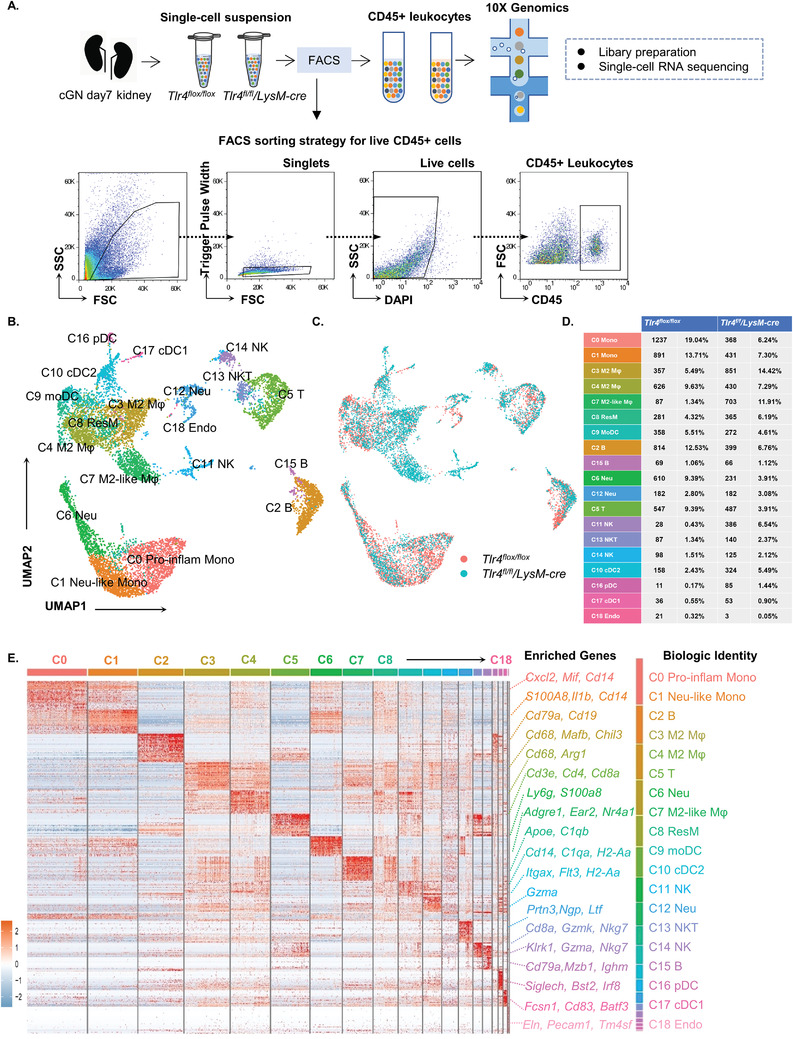
Single‐cell RNA sequencing of renal CD45+ leukocytes identifies 19 distinct immune cell subpopulations in *Tlr4^flox/flox^* and *Tlr4^fl/fl^/LysM‐cre* mice with anti‐GBM cGN. A) Graphical illustration of the experimental setup. Samples were isolated from kidney of *Tlr4^flox/flox^* and *Tlr4^fl/fl^/LysM‐cre* mice at day 7 after anti‐GBM cGN induction in duplicates. Kidneys were digested into single‐cell suspension and live CD45+ cells were FACS‐sorted and loaded cells for scRNA‐seq. B) Nonlinear dimensionality reduction Uniform Manifold Approximation (UMAP) visualization of 12 399 renal CD45+ immune cells identified 19 different clusters after unsupervised clustering in *Tlr4^flox/flox^* and *Tlr4^fl/fl^/LysM‐cre* group, respectively. Each point depicts a single cell, colored according to cluster designation. C) UMAP visualization of CD45+ immune cell populations colored in accordance of group. D) Summary of proportion of assigned cell types in *Tlr4^flox/flox^* and *Tlr4^fl/fl^/LysM‐cre* group, respectively. E) Heatmap showing the top 50 most differentially upregulated genes in each cluster identified through unsupervised clustering of renal immune cells. Light blue indicates lower expression; Red indicates higher expression. Average expression (avg. exp) scale is shown on the right. Known cell type markers strongly and specifically associated with major cell types are shown on the right. Pro‐inflam Mono: pro‐inflammatory monocyte, Neu‐like Mono: neutrophil‐like monocyte, M*φ*: macrophage, ResM: resident macrophage, moDC: monocyte‐derived dendritic cell, B: B cell, Neu: neutrophil, T: T cell, NK: natural killer cell, NKT: natural killer T cell, cDC1: type 1 conventional dendritic cell, cDC2: type 2 conventional dendritic cell, pDC: plasmacytoid dendritic cell, Endo: endothelial cell.

Using 10× Genomics scRNA‐seq platform, we characterized 12 399 fluorescence‐activated cell sorting (FACS) isolated live CD45^+^ immune cells (6498 cells from *Tlr4^flox/flox^* mice and 5901 cells from *Tlr4^fl/fl^/LysM‐cre* group) from anti‐GBM cGN induced kidney at day 7 after quality control (**Figure** **1**A). Diverse immune cell landscape was mapped via non‐linear dimensionality reduction on Uniform Manifold Approximation and Projection (UMAP) plot for *Tlr4^flox/flox^* and *Tlr4^fl/fl^/LysM‐cre* group after integration analysis (Figure 1B,C). Resolution tree of clusters with an additional subcluster layer was tested for validating the legitimacy of the cluster resolution (Figure S1, Supporting Information). As a result, nineteen distinct clusters of CD45^+^ immune cells were identified by Seurat clustering algorithm (Figure 1B). The cell type identification for each cluster was decided based on significant expression of well‐characterized marker genes (Figure 1E). As expected, macrophages/monocytes (Cluster 0, 1, 3, 4, 7, 8, and 9) were the most predominant immune cells (59.3% of total CD45^+^ immune cells), followed by B cells (Clusters 2 and 15), neutrophils (Cluster 6 and 12), and T cells (Clusters 5). Nature killer (NK) cells appeared in two distinct clusters (Clusters 11 and 14), while Cluster 13 was recognized as NKT cells (*Cd8a^+^*, *Nkg7^+^*). Cluster 10, 16, and 17 corresponded to dendritic cells. Endothelia cells, implicated in glomerular crescent formation in the past in cGN,^[^ ^12^ ^]^ also appeared in Cluster 18. The cluster numbers corresponded to the ranking of relative cell abundance of each cluster (Figure 1D).

### 2.2 Deletion of Myeloid‐TLR4 Altered Macrophage Functional Heterogeneity toward Anti‐Inflammatory Dominant Configuration

**Figure 2:**
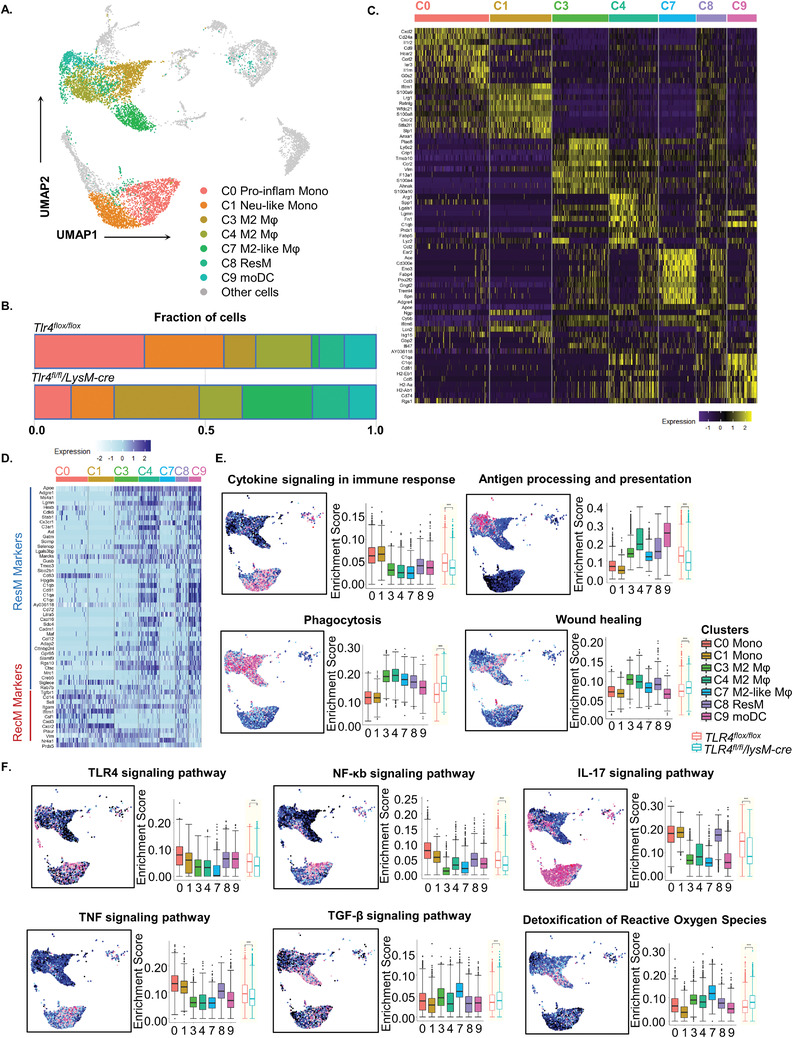
Single‐cell RNA sequencing reveals macrophage heterogeneity in anti‐GBM cGN in *Tlr4^flox/flox^* and *Tlr4^fl/fl^/LysM‐cre* mice. A) UMAP visualization of seven distinct kidney macrophage/monocyte clusters (Cluster 0, 1, 3, 4, 7, 8, and 9), in *Tlr4^flox/flox^* and *Tlr4^fl/fl^/LysM‐cre* integrated transcriptome profile at day 7 post anti‐GBM cGN induction, colored according to cluster designation. B) Bar plot showing the proportions of cells among macrophage/monocyte cell populations, colored according to cluster designation. C) Heatmap showing the top 10 most differentially upregulated genes in each monocyte/macrophage subcluster identified through unsupervised clustering. Expression scale is shown on the bottom. D) Heatmap for renal resident and recruited macrophage markers expression among macrophage/monocyte subpopulations. Expression scale is shown on the bottom. Colored UMAP plots highlighted cells with activated gene sets expression in E) antigen processing and presentation, wound healing, phagocytosis and cytokine signaling in immune response, and F) TLR4, NF‐*κ*B, IL‐17, TNF, TGF‐*β* and detoxification of reactive oxygen species signaling among macrophages/monocytes based on AUC scores. Each point is depicted a single cell. Cells with indicated signaling activation were colored in shades of pink‐red, and those without signaling activation were colored in black‐blue. Boxplots on the right statistically compare AUC enrichment scores of genes annotated for relative pathways between the seven macrophages/monocytes clusters, and between *Tlr4^flox/flox^* and *Tlr4^fl/fl^/LysM‐cre* group. The complete lists of genes used to compute enrichment are according to KEGG: mmu04612 [antigen processing and presentation], GO:0042060 [wound healing], GO:0006909 [phagocytosis], Reactome: R‐MMU‐1280215 [Cytokine signaling in immune response], GO:0050729 [TLR4 signaling pathway], KEGG: mmu04064 [NF‐*κ*B signaling pathway], KEGG: mmu04657 [IL‐17 signaling pathway], KEGG:mmu04668 [TNF signalling pathway], KEGG: mmu04350 [TGF‐*β* signaling pathway] and Reactome: R‐MMU‐3299685 [detoxification of reactive oxygen species]. *p < 0.05, **p < 0.05, *** *p* < 0.001 versus corresponding *Tlr4^flox/flox^*. Pro‐inflam Mono: pro‐inflammatory monocyte, Neu‐like Mono: neutrophil‐like monocyte, Mφ: macrophage, ResM: resident macrophage, moDC: monocyte‐derived dendritic cell.

In response to crescentic glomerulonephritis, seven transcriptionally distinct subpopulations of macrophages/monocytes were identified, including two groups of *Cd14^+^* monocytes (Cluster 0, 1) and five clusters of *Adgre1^+^ (F4/80)* macrophages (Cluster 3, 4, 7, 8, 9) (**Figure** **2**A). A remarkable heterogeneous cell distribution of *Tlr4^flox/flox^* and *Tlr4^fl/fl^/LysM‐cre* cells was primarily identified in individual macrophage/monocyte subset (Figures 1B–D and 2B). Myeloid‐TLR4 deletion resulted in a sharp decrease in Cluster 0 *Cd14^hi^Mif^hi^* monocytes (77.07% from *Tlr4^flox/flox^* and 22.93% from *Tlr4^fl/fl^/LysM‐cre* group) and Cluster 1 *Cd14^hi^ Il1b^hi^ S100a8^hi^* granulocyte monocyte progenitors (GMPs)‐derived neutrophil‐like monocytes (67.40% from *Tlr4^flox/flox^* and 32.60% from *Tlr4^fl/fl^/LysM‐cre* cells), whereas promoting a sharp three‐ and ninefold increase in Clusters 3 (*Adgre1^+^ Chil3^+^*) and Cluster 7 (*Adgre1^+^ Nr4a1^+^ Ear2^+^*) macrophages, respectively, in *Tlr4^fl/fl^/LysM‐cre* group, compared to the littermate control. We also found that Cluster 0 and 1 pro‐inflammatory monocytes expressed the highest level of TLR4, compared to the rest five macrophage subsets (Figure S2, Supporting Information). This may explain why the deletion of myeloid‐TLR4 primarily inhibited the infiltration of these two proinflammatory monocyte subsets, rather than the rest anti‐inflammatory or resident macrophages. Differential expression analysis for detection of top ranked differentially expressed genes (DEGs) further illustrated well‐distinguished transcriptional heterogeneity among individual subtype (Figure 2C).

Origin of each macrophage/monocyte subpopulation was characterized by examining expression of known resident and infiltrated macrophage marker genes.^[^ ^13^, ^14^, ^15^ ^]^ As a result, resident macrophage signatures, such as *Mrc1(Cd206)*, *Cd74*, *Cd81*, *C1qa*, *C1qc* were highly expressed in Clusters 4, 8, and 9 macrophages (Figure 2D). Consistent with our previous findings,^[^ ^11^ ^]^ deletion of myeloid TLR4 did not alter the proportion of the tissue resident macrophages (Cluster 4, 8, and 9) between *Tlr4^flox/flox^* and *Tlr4^fl/fl^/LysM‐cre* anti‐GBM cGN mice group (Figure 1D). On the other hand, recruited monocyte/macrophages, identified by expressing *Cd14*, *Sell*, *Cxcr2*, *Ifitm1*, were significantly upregulated in Cluster 0 and 1, which markedly demonstrating the M1 proinflammatory macrophage phenotype and were largely suppressed in the diseased kidney when myeloid‐TLR4 was deleted (Figure 1D and 2D–F).

Functional heterogeneity among individual macrophage/monocyte cluster was further investigated using AUCell algorithm (Area Under the Curve [AUC]) based gene set enrichment analysis (GSEA)^[^ ^16^ ^]^ (Figure 2E,F). As a result, Cluster 0 and 1 monocytes demonstrated essentially enriched cytokine signaling in immune response, compared to the rest macrophage populations (Figure 2E). Genes involved in pro‐inflammatory signaling pathways, including TLR4 and its downstream NF‐*κ*B and TNF signaling were greatly activated in Cluster 0 and 1 cells (Figure 2F), suggesting the pro‐inflammatory phenotype of these two clusters being the monocyte subsets. Another interesting finding was that IL‐17 signaling was also greatly activated in these two pro‐inflammatory monocyte subgroups (Figure 2F). IL‐17 is a well‐known proinflammatory cytokine mainly produced by Th17 cells, which plays an important role in promoting immune injury during autoimmune and infectious diseases. Several studies have reported that activation of IL‐17 signaling can promote macrophage recruitment,^[^ ^17^ ^]^ polarize macrophages toward a proinflammatory transcriptome,^[^ ^18^ ^]^ or even enhance the expression of TLR4 in macrophages.^[^ ^19^ ^]^ By using scRNA‐seq, our study firstly demonstrated the activation of IL‐17 signaling in TLR4‐dependent proinflammatory macrophage subsets at early stage of experimental anti‐GBM cGN, indicating the IL‐17 mediated downstream cascade may be another potential mechanism in anti‐GBM cGN. Compared to Cluster 0, Cluster 1 monocytes uniquely expressed neutrophil‐like gene signatures, such as *S100a8, S100a9 and Lrg1*, while lacking of *Ly6g* expression (Figure 2C and Figure S3, Supporting Information), suggesting their potential identity as GMPs‐derived neutrophil‐like monocytes.^[^ ^20^ ^]^ Cluster 9 cells also highly expressed *Cd14* monocyte gene, with its transcriptome profile uniquely enriched in antigen processing and presentation signaling (Figure 2E and Figure S3, Supporting Information), thus disclosing their antigen presenting capacities as monocyte‐derived dendritic cells (moDCs).^[^ ^21^, ^22^, ^23^ ^]^ The Cluster 3 infiltrated (*Ly6c2^high^, Ccr2^+^*) and Cluster 4 resident (*Ly6c2^low^, Ccr2^low^*) M2 macrophages were distinctively enriched for wound healing signaling (Figure 2E).

Interestingly, we identified a novel and unique TLR4‐dependent Cluster 7 *Nr4a1^+^ Ear2^+^* macrophages, with the expression profile and functional activities distinguished from any known macrophage phenotypes including M1 or M2. It was the most affective macrophage cluster in response to the myeloid‐TLR4 deletion, with a 9‐fold increase in *Tlr4^fl/fl^/LysM‐cre* mice with anti‐GBM cGN when compared to the *Tlr4^flox/flox^* littermates. Cluster 7 shared similar anti‐inflammatory signatures with Cluster 3 and 4 M2 macrophages, with enriched wound healing and phagocytosis signals and markedly suppressed M1‐related pro‐inflammatory signaling (Figure 2F). However, they were also well distinguished from the two M2 macrophage clusters by uniquely expressing genes enriched in detoxification of reactive oxygen species (ROS) and TGF‐*β* signaling (Figure 2E). In addition, there were 481 DEGs identified in Cluster 7 macrophages, compared to those M2 macrophages from Cluster 3 and 4 via differentially expression analysis (Table S1, Supporting Information). In comparison, Cluster 7 macrophages demonstrated enhanced negative regulation of immune response and cytokine production, as well as immunoregulatory interactions between a lymphoid and nonlymphoid cell signal, and inhibited neutrophil degranulation, leukocyte chemotaxis, phagosome and lysosome signals (Figure S4, Supporting Information).

As the *LysM* promoter‐driven *Cre* recombinase also results in functional deficiency of TLR4 in neutrophils, we could not exclude the potential role of neutrophils‐specific TLR4 in anti‐GBM cGN. Based on unbiased clustering, differential expression and enrichment analysis of our scRNA‐seq profile, we did find that deletion of myeloid‐TLR4 not only inhibited Cluster 6 pro‐inflammatory neutrophil infiltration, but also suppressed the neutrophil‐mediated inflammatory cytokine expression (Figure 1D and Figure S5, Supporting Information). However, several studies have already reported that neutrophil infiltration is only the early and transient event during anti‐GBM cGN.^[^ ^1^, ^24^ ^]^ Moreover, only a small population of neutrophils (accounting for 9.7% of total CD45^+^ leukocytes) were identified both in the present scRNA‐seq data set and in our previous study in anti‐GBM cGN mouse kidney.^[^ ^11^ ^]^ Thus, it is likely that macrophages are the major cell type that plays a critical role in anti‐GBM cGN.

Regardless of clusters, deletion of myeloid‐TLR4 significantly suppressed pro‐inflammatory cytokine signaling, such as NF‐*κ*B, IL‐17, and TNF signaling pathways, weakened the antigen presenting capacity, and gravely enhanced anti‐inflammatory signaling, including wound healing and TGF*β* signaling within overall renal macrophages/monocytes at day 7 post anti‐GBM cGN (Figure 2E,F).

### 2.3 Deletion of Myeloid‐TLR4 Facilitated Macrophage Differentiation from Pro‐Inflammatory toward Anti‐Inflammatory Phenotype in Anti‐GBM cGN

**Figure 3:**
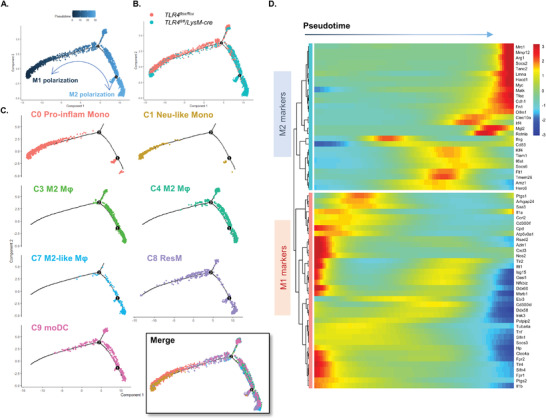
Pseudotime analysis uncovered TLR4‐dependent regulation on macrophages/monocytes differentiation in anti‐GBM cGN in *Tlr4^flox/flox^* and *Tlr4^fl/fl^/LysM‐cre* mice. Visualization of cell trajectories of A) entire monocyte/macrophage, or B) split by *Tlr4^flox/flox^* and *Tlr4^fl/fl^/LysM‐cre* group and C) Seurat clusters separately revealed distinct distribution of macrophage/monocyte subsets into each activation state. D) Heatmap revealed pseudotemporal expression pattern of clustering genes among macrophage/monocyte populations in anti‐GBM cGN. Pseudotime‐dependent DEGs following the similar kinetic trends are categorized into the same cluster. Expression scale is shown on the right.

To uncover the hypothetical TLR4‐dependent regulation on macrophage/monocytes differentiation in crescentic glomerulonephritis, we re‐clustered these populations (Cluster 0, 1, 3, 4, 7, 8, and 9) to construct single cell–based differentiation trajectory in our scRNA‐seq data (**Figure** **3**A). Seurat‐defined clusters were superimposed on a pseudotime trajectory produced by the Monocle 2 algorithm.^[^ ^25^, ^26^ ^]^ As shown in Figure 3B, *Tlr4^flox/flox^* macrophage/monocytes primarily aggregated in the left half of the major trajectory branch, while those with TLR4 deletion were broadly distributed toward the end of the trajectory path on the right‐side trajectory. Specifically, two pro‐inflammatory monocytes: Cluster 0 (*Cd14^+^ Mif^hi^*) and Cluster 1 (*Cd14^+^ Il1b^hi^*), predominantly from *Tlr4^flox/flox^* group, were located at the left start point of trajectory branch. Meanwhile, Cluster 7 and 3 macrophages which were enriched from *Tlr4^fl/fl^/LysM‐cre* macrophages, together with Cluster 4 resident M2 macrophages, were dominantly gathered at the terminal trajectory. The Cluster 8 resident macrophages and Cluster 9 moDCs straddled among all branches across the pseudotime space, indicating their transfer status at day 7 post anti‐GBM cGN (Figure 3C).

To further understand the genetic characteristic of macrophage/monocyte clusters distributed alongside the pseudotime trajectory, DEGs that co‐vary alongside pseudotime were visualized by Monocle2. As illustrated in Figure 3D, the M1 macrophage‐related signatures, such as *Nos2, Il1b, Tlr4, Tlr2*, etc., notably increased both in density and expression level at the beginning of pseudotime trajectory, where *Tlr4^flox/flox^* macrophages/monocytes were mainly distributed. While M2 macrophage highly expressed gene modules, including *Arg1, Mrc1, Retnla*, etc., were greatly upregulated at the end stage of the cell trajectory, where *Tlr4^fl/fl^/LysM‐cre* macrophages/monocytes preferentially located. Moreover, to validate that the direction of macrophage/monocyte transition alongside the reconstructed trajectory in Figure 3A–C is from the left start M1 to the right terminal M2 genotype, we conducted robust quantification of representative M1 and M2 marker genes expression in every single cell, and tracked the M1 to M2 transcriptome changes over pseudo time (Figure S6, Supporting Information). Dot plots illustrated the expression patterns transition from M1 to M2 genotype in individual cells from different clusters over pseudo time. Moreover, in combination of Figure 3B, the results in Figure S6 (Supporting Information) further indicated that deletion of TLR4 significantly suppressed the M1 but increased the M2 transcription.

Based on the pseudotime cell developmental trajectory reconstruction, we uncovered the transitional associations between diverse monocyte/macrophage subpopulations under regulation of TLR4. Pro‐inflammatory *Cd14^+^Mif^hi^* Cluster 0 monocytes and *Il1b^hi^ S100a8^+^* Cluster 1 neutrophil‐like monocytes were likely to differentiate into *F4/80^+^* M2‐like macrophages (Cluster 3, 4, 7) in the inflamed kidney with anti‐GBM cGN when myeloid‐TLR4 was disrupted. More importantly, we shed light upon the essential role of TLR4 in cell fate and polarization decision at the transcriptome level, that deletion of myeloid‐TLR4 greatly enhanced the macrophage differentiation from pro‐inflammatory M1‐like monocytes toward M2‐like anti‐inflammatory macrophages (Figure 3).

### 2.4 Single‐Cell RNA‐seq Characterized Myeloid‐TLR4 Dependent and Cell‐Type Specific TF‐Centered Gene Regulatory Networks

**Figure 4:**
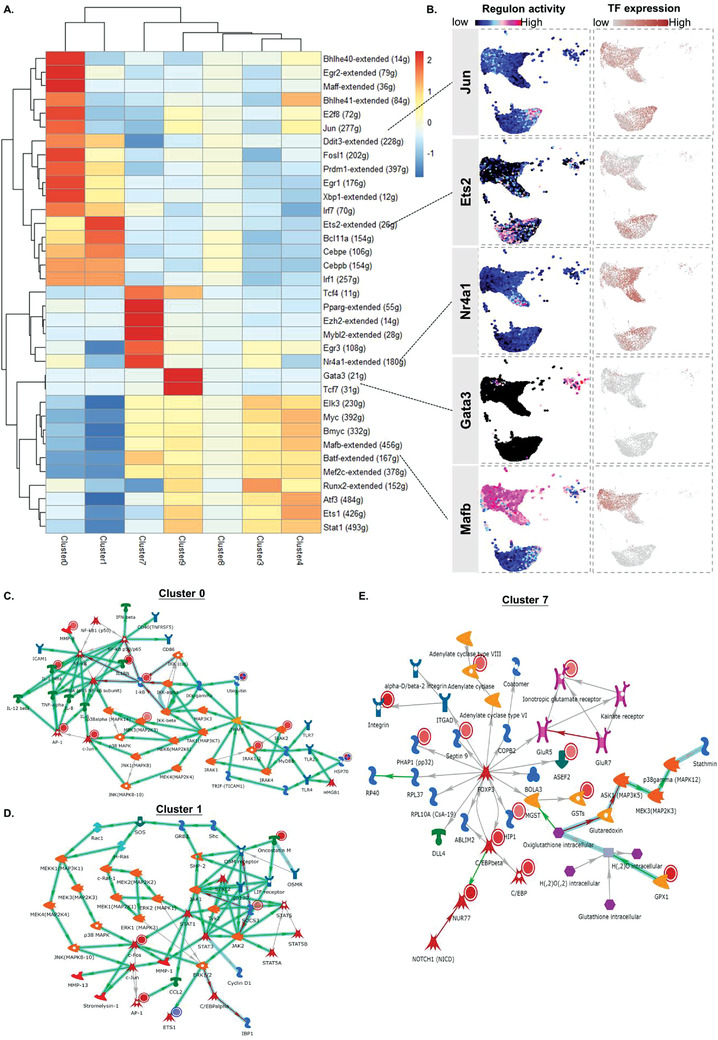
Macrophage/monocyte specific gene regulatory landscape in experimental anti‐GBM cGN in *Tlr4^flox/flox^* and *Tlr4^fl/fl^/LysM‐cre* mice. A) Heatmap of cell type‐specific top ranked TFs regulons in each macrophage/monocyte cluster, as inferred by SCENIC algorithm. Enrichment score scale is shown on the right‐side legend. B) UMAP depiction of cell‐type specific top regulon activity (“high‐pink/red,” “low‐dark/blue”) and TF gene expression (brown scale) of exemplary regulons for Jun (Cluster 0 pro‐inflammatory monocyte), Ets2 (Cluster1 neutrophil‐like monocyte), Nr4a1 (Cluster7 anti‐inflammatory macrophage), Gata3 (Cluster9 moDC), Mafb (Cluster 3 and 4 M2 macrophage). Metacore analysis revealed top GRN reconstruction in C) Cluster 0, D) Cluster 1, and E) Cluster 7. Unbiased regulatory networks are generated based on shortest direct interactions algorithms via MetaCore analytical program according to the high confidential DEGs (*p* < 0.01) found in each macrophage/monocyte subclusters. Involved DEGs from the cell type‐specific data set are highlighted by a solid circle at the upper right‐hand side whose relative expression level is represented by different intensity in red color.

To identify the cell‐type specific TFs and TF‐centered gene regulatory networks (GRNs), which responsible for macrophage heterogeneity in anti‐GBM cGN, we performed Single‐Cell rEgulatory Network Inference and Clustering (SCENIC)^[^ ^16^ ^]^ in combination of Metacore pathway enrichment analysis.^[^ ^27^ ^]^ In brief, Co‐expression modules between TFs and candidate target genes were firstly inferred, followed by identification of GRNs with direct binding to the activated TFs in every single cell using GENIE3 and RcisTarget. Top GRNs enriched in each cluster were constructed based on the shortest path algorithm interaction networks found by MetaCore analytical suite. As shown in **Figure** **4**A, SCENIC results indicated strong enrichment in *Bhlhe40*, *Egr2*, *Maff*, *E2f8*, *Jun*, *Fosl1*, *Prdm1*, *Ddit3* regulon activity in Cluster 0 monocytes, and in pro‐inflammatory *Cebpe*, *Cebpb*, *Ets2*,^[^ ^28^ ^]^ *Irf1*,^[^ ^29^ ^]^ and *Bcl11a*‐centric GRNs in Cluster 1 neutrophil‐like monocytes, respectively. Conversely, *Nr4a1*, together with immunotolerance‐related *Tcf4*,^[^ ^30^ ^]^ and M2 macrophage polarization‐associated *Pparg* ^[^ ^31^ ^]^ regulons were specifically enriched in Cluster 7 macrophages. The other M2 macrophage groups Cluster 3 and 4 shared the similar candidate TFs regulons such as *Myc*, *Bmyc*, *Mafb*, *Batf*, with the *Runx2* regulon demonstrating stronger enrichment in Cluster 3, and *Stat1, Ets1* regulons scored higher in Cluster 4. For Cluster 9 moDCs, SCENIC analysis suggested *Gata3‐* and *Tcf7*‐dependent GRNs as the mostly enriched regulatory networks. Examples of representative regulon activity and corresponding TF expression are depicted in Figure 4B.

Furthermore, Metacore analysis identified two TLR4‐dependent regulatory networks “I‐kB/AP‐1/IL‐1*β*/c‐Jun/MEK3 (MAP2K3)” and “c‐Fos/SOCS3/Oncostatin M/AP‐1/STAT3” as the predominant GRNs containing the highest numbers of DEGs and most outstanding significance in Cluster 0 (Figure 4C) and Cluster 1 (Figure 4D) monocytes, respectively, emphasizing the pivotal role of myeloid‐TLR4 in pro‐inflammatory monocytes polarization and infiltration during renal inflammation in anti‐GBM cGN. Interestingly, for Cluster 7 macrophages that derived by TLR4 deficiency, a Foxp3‐centric “Foxp3/CEBPb/Nur77(Nr4a1)” network turned out to be the mostly enriched signalling pathway (Figure 4E), indicating the immune regulatory role of this unique macrophage subtype in anti‐GBM cGN.

### 2.5 Deletion of Myeloid‐TLR4 Induced a Novel Anti‐Inflammatory Macrophage Subset by Activation of NUR77(Nr4a1)/Ear2 Feedback Signaling

Our scRNA‐seq profile illustrated that deletion of myeloid‐TLR4 resulted in an increase in a novel anti‐inflammatory macrophage subset Cluster 7 (*Adgre1^+^ Nr4a1^hi^ Ear2^hi^*) in experimental anti‐GBM cGN (Figures 1 and 2), which was clearly and constantly distinguished from any other clusters throughout the clustree from low (0.1) to high (1) resolution (Figure S1A, Supporting Information), with relatively high sc3 stability score (Figure S1B, Supporting Information). To further confirm the presence of the Cluster 7 *Nr4a1^+^Ear2^+^* macrophage subgroup, we excluded the Cluster 0, Cluster 1 monocytes and Cluster 9 moDC, and re‐clustered the rest macrophage subsets (Cluster 3, 4, 7, and 8) for identification of *Nr4a1^+^Ear2^+^* macrophages by unbiased clustering with Seurat. As expected, visualization of co‐expression also illustrated the *Nr4a1^+^Ear2^+^* macrophages as an independent cluster of cells (Figure S7, Supporting Information). We therefore confirmed the presence of the Cluster 7 *Nr4a1^+^Ear2^+^* macrophage subgroup at day 7 post anti‐GBM cGN at transcriptome level.

**Figure 5:**
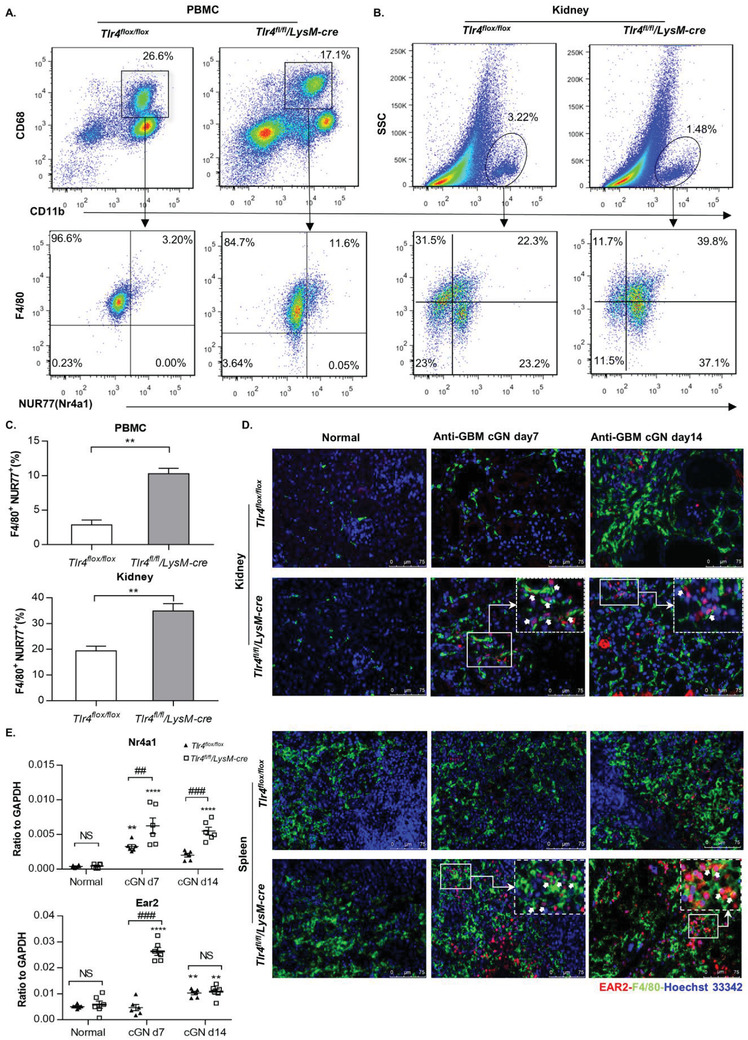
Identification of the TLR4‐dependent novel Cluster 7 (*Nr4a1^+^ Ear2^+^*) anti‐inflammatory macrophage phenotype in anti‐GBM cGN in *Tlr4^flox/flox^* and *Tlr4^fl/fl^/LysM‐cre* mice. A) Flow cytometry analysis for PBMC at day 7 post experimental anti‐GBM cGN. For upper panel: total CD11b^+^ CD68^+^ cells, lower panel: F4/80^+^ NUR77 (NR4A1)^+^ macrophages. B) Flow cytometry analysis for kidney single cell suspension, for upper panel: total CD11b^+^ myeloid cells, lower panel: F4/80^+^ NUR77/NR4A1^+^ macrophages. C) Statistical data of (A) and (B). Data represent the mean ± SEM for groups of at least three mice. **P < 0.01 versus corresponding *Tlr4^flox/flox^*. D) Representative immunofluorescence images of EAR2 (red), F4/80 (green), and Hoechst 33342 (blue) in the kidney and spleen of normal control and at day 7 and day 14 post anti‐GBM cGN in *Tlr4^flox/flox^* and *Tlr4^fl/fl^/LysM‐cre* mice. Arrows demonstrate Cluster 7 macrophage phenotype by co‐expressing EAR2 (red) and F4/80 (green). E) *Nr4a1* and *Ear2* gene expression in normal kidney and kidney at day 7 and day 14 post anti‐GBM cGN. Data represent the mean ± SEM for groups of at least three mice. **P < 0.01, ****P < 0.0001 versus Normal; NS: no significant difference, ##*P* < 0.01, ###*P* < 0.001 versus corresponding *Tlr4^flox/flox^*.

To validate the regulatory role of TLR4 in this novel macrophage phenotype, we firstly tested their marker genes (*Nr4a1* and *Ear2*) expression in kidneys after anti‐GBM cGN induction. Consistent with our scRNA‐seq findings, flow cytometry analysis confirmed that deletion of myeloid‐TLR4 led to a threefold and doubled increase in F4/80^+^NUR77 (Nr4a1) ^+^ macrophage population in PBMC and diseased kidney, respectively, at day 7 post anti‐GBM cGN (**Figure** **5**A–C). Immunofluorescence staining also demonstrated that deletion of myeloid‐TLR4 markedly promoted F4/80^+^EAR2^+^ macrophages infiltration in both spleen and diseased kidney of *Tlr4^fl/fl^/LysM‐cre* mice, compared to their *Tlr4^flox/flox^* littermates (Figure 5D). Similar changes of *Nr4a1* and *Ear2* overall expression in entire kidneys in vivo between *Tlr4^flox/flox^* and *Tlr4^fl/fl^/LysM‐cre* groups were also observed by real‐time PCR (Figure 5E).

**Figure 6:**
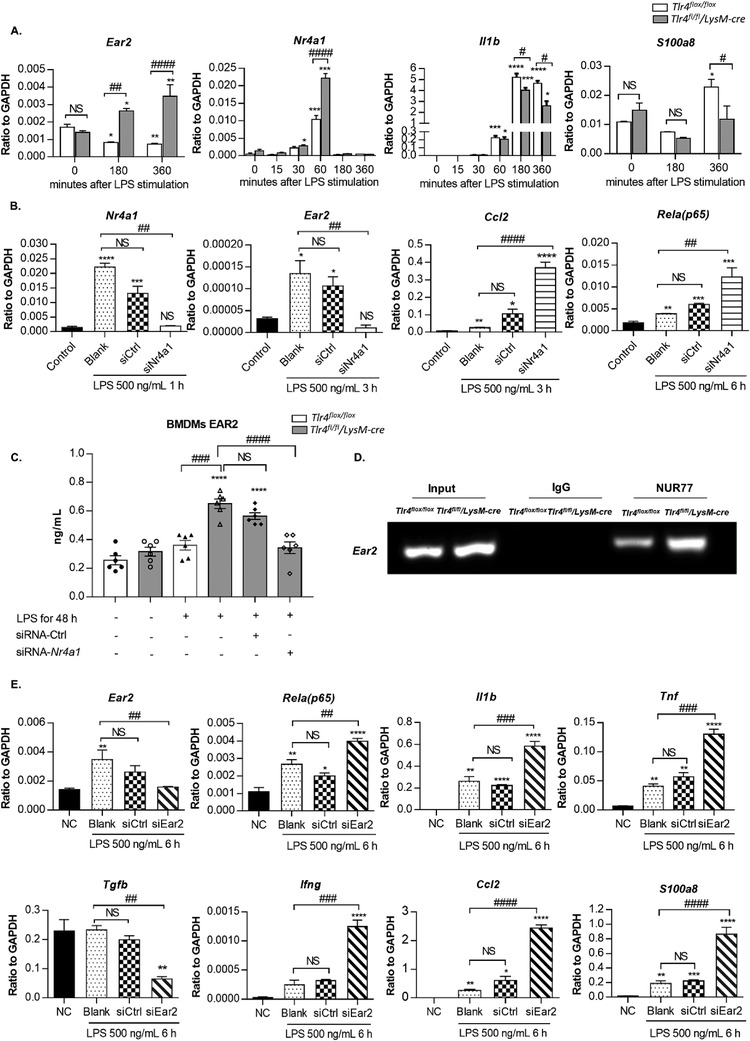
A novel Nr4a1/Ear2 axis mediated anti‐inflammatory signaling in TLR4 deficient BMDMs. A) Different macrophage/monocyte clusters’ maker genes expression in LPS‐stimulated BMDMs in vitro between *Tlr4^flox/flox^* and *Tlr4^fl/fl^/LysM‐cre* group. B) Gene expression in BMDMs, with or without *Nr4a1* siRNA (siNr4a1) or Ctrl siRNA (siCtrl) pre‐treatment, under LPS stimulation for 6 h. C) ELISA analysis of BMDMs EAR2 protein expression with or without siNr4a1 or siCtrl pre‐treatment, under 500 ng mL^−1^ LPS stimulation for 48 h. D) Direct binding of Nr4a1 subunit on *Ear2* promoter region in BMDM under LPS stimulation shown by ChIP assay. E) Gene expression in BMDMs, with or without *Ear2* siRNA (siEar2) or siCtrl pre‐treatment, under LPS stimulation for 6 h.

It has been reported that, TLR4 activation by LPS stimulation can significantly induce M1 response in macrophages,^[^ ^32^ ^]^ the later can then induce remarkable renal injury in experimental anti‐GBM cGN.^[^ ^33^ ^]^ Our previous study also showed that deletion of myeloid‐TLR4 in *Tlr4^fl/fl^/LysM‐cre* mice significantly increased M2 macrophage proportion while significantly inhibiting the M1 macrophage infiltration in the diseased kidney.^[^ ^11^ ^]^ However, the mechanisms whereby deletion of myeloid‐TLR4 promotes anti‐inflammatory macrophage differentiation during anti‐GBM cGN remain largely unclear and need to be further investigated. The nuclear receptor Nur77 (*Nr4a1*) has been reported as a TLR4‐dependent feedback effector of NF‐*κ*B.^[^ ^34^ ^]^ It can be activated in human and mouse macrophages in response to LPS stimulation,^[^ ^34^, ^35^ ^]^ and in turn abolished macrophage inflammatory response via inhibition of NF‐*κ*B signaling.^[^ ^36^ ^]^ However, the underlying mechanism of NUR77‐mediated anti‐inflammatory activity in macrophages remains largely unknown. Finding of this novel *Nr4a1^+^ Ear2^+^* anti‐inflammatory macrophage subtype in our scRNA‐seq analysis strongly suggested *Ear2* as a potential downstream target of *Nr4a1*, contributing to the limitation of macrophage inflammatory response. To further validate our hypothesis, we conducted in vitro study in LPS‐stimulated bone marrow derived macrophages (BMDMs) of *Tlr4^flox/flox^* and *Tlr4^fl/fl^/LysM‐cre* mice. Upon LPS stimuli, deletion of TLR4 significantly increased both *Ear2* and *Nr4a1* expression in BMDMs, compared to the *Tlr4^flox/flox^* group. On the contrary, pro‐inflammatory *Il1β* and *S100a8* that highly expressed in Cluster 0 and 1 monocytes were significantly suppressed in *Tlr4^fl/fl^/LysM‐cre* BMDMs (**Figure** **6**A). More importantly, silencing of *Nr4a1* by siRNA (siNr4a1) resulted in significant downregulation of *Ear2*, both at mRNA and protein level in LPS‐stimulated *Tlr4^fl/fl^/LysM‐cre* BMDMs, as evaluated via real‐time PCR and ELISA, respectively (Figure 6B,C), indicating that EAR2 expression is under regulation of *Nr4a1* in BMDMs. Consequently, TLR4 deficiency‐driven inhibition of *Ccl2* and *p65* were also counteracted due to *Nr4a1* suppression (Figure 6B).

By chromatin immunosuppression (ChIP) assay, we demonstrated that deletion of TLR4 significantly enhanced the physical binding of NUR77 (Nr4a1) protein on the 3′UTR region of the *Ear2* gene in BMDM under LPS stimulation (Figure 6D). In accordance with scRNA‐seq profile, the *Nr4a1^+^Ear2^+^* macrophage phenotype is transcriptionally distinguished from the known M1 and M2 phenotype. Therefore, instead of testing the M1/M2 signatures, to reveal the anti‐inflammatory effect of the TLR4‐dependent Nr4a1/Ear2 axis on macrophages, we directly examined the pro‐inflammatory *Rela*(*p65)*, *Il1β*, *Tnf*, *Ifng*, *Ccl2* and *S100a8* genes and the anti‐inflammatory *Tgfb* expression by silencing the *Ear2* gene expression in *Tlr4^fl/fl^/LysM‐cre* BMDMs. Functionally, silencing of *Ear2* by siRNA (siEar2) significantly increased the macrophage pro‐inflammatory activity, which was suppressed by deleting TLR4 in LPS‐stimulated *Tlr4^fl/fl^/LysM‐cre* BMDMs (Figure 6E).

Taken together, our results clearly demonstrated the important role of TLR4‐dependent Nr4a1/Ear2 axis in limiting macrophage pro‐inflammatory response while promoting the anti‐inflammatory phenotype both in vivo in anti‐GBM cGN and in vitro in BMDMs.

## 3 Discussion

Macrophages and toll‐like receptors are the well‐known versatile players during renal inflammation and fibrosis.^[^ ^4^, ^37^, ^38^, ^39^ ^]^ In the present study, by using scRNA‐seq, in combination of cell developmental trajectory reconstruction and transcription factor‐centered motif enrichment analysis, we profiled the unbiased renal immune landscape and decoded the mechanism of myeloid‐TLR4 dependent immune regulation in anti‐GBM cGN. We uncovered the diverse macrophage phenotypes in anti‐GBM cGN under the tight regulation of myeloid‐TLR4. We found that deletion of myeloid‐TLR4 promoted the bone‐marrow‐derived macrophages, but not resident macrophages differentiation from the M1 proinflammatory to the M2 anti‐inflammatory phenotype to exhibit the renoprotective effect on anti‐GBM cGN. The shift of these transcription profiles from M1 to M2 in *Tlr4^fl/fl^/LysM‐cre* mice with anti‐GBM cGN were consistent with previous findings that deletion of TLR4 results in one to two‐fold increase in F4/80^+^ CD206^+^ M2 macrophages while inhibiting about 50% of F4/80^+^ iNOS^+^ M1 macrophages infiltration and expression of renal proinflammatory cytokines such as IL‐1*β* and MCP‐1 at day 7 and 14 post anti‐GBM cGN.^[^ ^11^ ^]^ Most significantly, we discovered that deletion of myeloid‐TLR4 induced a novel and unique reparative macrophage population identified by a marked expression of two transcriptomes*Nr4a1* and *Ear2* with a significant upregulation of the anti‐inflammatory and tissue repair related pathways involving the detoxification of reactive oxygen species, phagocytosis, and TGF‐*β* signalling. Thus, the identification of Nr4a1/Ear2‐dependent anti‐inflammatory macrophage population was highly significant both scientifically and clinically since it may imply that the induction of this anti‐inflammatory macrophage population via the Nr4a1/Ear2‐dependent mechanism may represent as a novel immunotherapy for anti‐GBM cGN. It is highly possible that the Nr4a1/Ear2‐expressing macrophages may have renoprotective effect in anti‐GBM cGN as evidenced by deletion of TLR4 inhibits progressive renal functional and histological injury, including glomerular crescentic formation in *Tlr4^fl/fl^/LysM‐cre mice*.^[^ ^11^ ^]^

The discovery of a TLR4‐dependent novel Nr4a1/Ear2 anti‐inflammatory macrophage population was the most important finding in this study. This was supported by the finding that deletion of myeloid‐TLR4 induced a novel population of macrophages (89% derived from *Tlr4^fl/fl^/LysM‐cre* cells) uniquely expressing *Nr4a1* and *Ear2*, which is transcriptomically distinguished from any known M1 and M2 phenotypes. Although the *Nr4a1^+^* monocyte/macrophage subsets have been identified in colon cancer as a monocyte subtype,^[^ ^40^ ^]^ in atherosclerotic aorta with antiatherogenic function,^[^ ^41^ ^]^ in hepatocellular carcinoma as myeloid‐derived suppressor cells (MDSCs),^[^ ^42^ ^]^ in normal mouse kidneys,^[^ ^13^ ^]^ and in ureteric obstruction as patrolling monocytes,^[^ ^43^ ^]^ the role and mechanisms that regulate the Nr4a1/Ear2‐expressing macrophages remain largely unclear. In this study, we characterized *Nr4a1^+^Ear2^+^* macrophages with increased detoxification of ROS and TGF*β* signal but suppressed pro‐inflammatory cytokine signalling and antigen presenting capacity. SCENIC and Metacore analysis further showed strong expression of transcription factor *Nr4a1* and strongly enriched Nr4a1‐ and Foxp3‐centered regulatory networks in this specific subset, indicating its immune regulatory role in anti‐GBM cGN. It is known that the nuclear receptor NUR77 is encoded by *Nr4a1* gene and functions as feedback target of NF‐*κ*B.^[^ ^34^, ^44^ ^]^ Although Ear2 is not previously found in macrophage‐specific Nr4a1‐centric gene network, in the present study, ChIP assay unexpectedly identified that *Ear2* served as a direct target gene of the orphan nuclear receptor family member protein NUR77 (Nr4a1). Functionally, inhibition of Nr4a1 significantly suppressed Ear2 production in TLR4‐deficient BMDMs in response to LPS stimulation. Thus, the transcriptional activation of the Nr4a1/Ear2‐axis may be a key mechanism through which deletion of myeloid‐TLR4 promoted anti‐inflammatory macrophage differentiation and immunosuppression in anti‐GBM cGN. This was confirmed by silencing macrophage specific *Nr4a1* or *Ear2* to reverse the anti‐inflammatory effects on TLR4 deficient BMDMs.

Another interesting finding in the present study was that TLR4 did not have much impact on resident macrophages compared to those recruited populations. This was supported by the findings that deletion of myeloid‐TLR4 did not significantly alter the resident macrophage population with comparable Clusters 4, 8, and 9 tissue macrophage population between *Tlr4^flox/flox^* and in *Tlr4^fl/fl^/LysM‐cre* mice. In contrast, the pro‐inflammatory state was provoked in *Tlr4^flox/flox^* mice with two major recruited monocyte/macrophage subsets (Cluster 0 and 1) that highly expressed genes involved in TLR4, NF‐*κ*B, TNF, and IL‐17 signaling pathways, whereas, deletion of myeloid‐TLR4 resulted in a two‐ to three fold decline of these two pro‐inflammatory macrophages. We therefore evidenced myeloid‐TLR4 as a key mediator for the recruitment of pro‐inflammatory macrophages via TLR4/MAPK/NF‐kB/AP1 signaling in anti‐GBM cGN. Moreover, using pseudotime trajectory analysis, we revealed the essential role of myeloid‐TLR4 in promoting macrophage differentiation toward M1 phenotype in anti‐GBM cGN at transcriptome level. However, additional cell lineage experiments are still needed to further confirm the myeloid‐TLR4 dependent regulation in macrophage transdifferentiation.

Increasing evidence shows that the use of scRNA‐seq analysis greatly contributes to the discovery of novel cell type, prognostic biomarkers, and cell type specific signaling pathway amenable to therapeutic targeting in immune‐related kidney disease. By aligning scRNA‐seq profile with GWAS database from clinical genetic studies, Park et al. identified the cell‐type specific contribution in diverse renal diseases.^[^ ^45^ ^]^ More recently, Yao et al. found a unique group of *S100a8/a9^+^* macrophage subset, similar to the Cluster 1 neutrophil‐like monocytes in our study, that were positively correlated with tissue injury in AKI by using scRNA‐seq. They further targeted the *S100a8/a9* by using small‐molecule inhibitors to successfully ameliorate renal damage and improve survival of the experimental AKI model, providing a promising and clinically relevant therapeutic strategy for human AKI.^[^ ^46^ ^]^ In this study, the combination of multiple complementary technologies enables us to identify novel TLR4‐dependent downstream target, the Nr4a1/Ear2 feedback axis, in anti‐GBM cGN. Although the therapeutic effect of NUR77 and EAR2 activation in anti‐GBM cGN remains unknown, earlier studies have reported that activation of NUR77 (Nr4a1) by using its agonist, such as cytosporone B or 6‐mercaptopurine, can significantly inhibit microglia‐mediated neuroinflammation, or prevent progression of pulmonary hypertension.^[^ ^47^, ^48^ ^]^ Moreover, deletion of NUR77 has been reported to polarize macrophage toward pro‐inflammatory phenotype, thus increasing atherosclerosis.^[^ ^21^ ^]^ Thus, it is also highly possible that the *Nr4a1/Ear2*‐expressing anti‐inflammatory macrophages could be a novel immunotherapy for immunologically mediated glomerulonephritis.

## 4 Experimental Section

### Experimental Anti‐GBM cGN Model in Mice

All animal experimental procedures in this study were carried with the approved protocol by the Animal Ethics Experimentation Committee at the Chinese University of Hong Kong (Ref. No. 18‐172‐MIS). Mice with myeloid‐specific TLR4 deletion (*Tlr4^fl/fl^/LysM‐cre*) were generated by crossing C57BL/6 mice bearing homozygous *loxP*‐flanked *Tlr4* (*Tlr4^flox/flox^*) (JAX stock number: 024872) and C57BL/6 mice with *lysozyme M* promoter‐driven *cre* (*LysM‐cre*) (JAX stock number: 004781) as previously described.^[^ ^11^ ^]^ As reported previously, compared to the *Tlr4^flox/flox^* littermates, the majority of TLR4 (>85%) was successfully and effectively deleted from macrophages in *Tlr4^fl/fl^/LysM‐cre* mice, as assessed at mRNA level by real time PCR and at protein level by flow cytometry, both in BMDMs and in the kidney macrophages with anti‐GBM cGN in *Tlr4^fl/fl^/LysM‐cre* mice.^[^ ^11^ ^]^

All animals were raised under a specific pathogen‐free condition at 25 °C with a normal 12‐h light and 12‐h dark cycle. Mice were allowed free access to standard food and sterilized water supplied by the animal unit in the Chinese University of Hong Kong.

Anti‐GBM cGN model was established in male *Tlr4^fl/fl^/LysM‐cre* and *Tlr4^flox/flox^* transgenic mice (C57/Bl6) at age 8–12 weeks in accordance with a well‐established protocol.^[^ ^11^, ^49^, ^50^ ^]^ In brief, mice were sensitized by subcutaneous injection of 2 mg of sheep globulin in 200 µL of Freund's complete adjuvant (Sigma Aldrich, St. Louis, MO) in each flank 5 d in advance, followed by anti‐GBM cGN initiation via intravenous administration of 5 mg of sheep anti‐mouse GBM IgG via tail vein. Groups of age‐ and gender‐matched *Tlr4^flox/flox^* and *Tlr4^fl/fl^/LysM‐cre* mice without disease induction were used as controls.

### Single‐Cell Preparation

Following systemic perfusion with cold PBS, four kidneys were harvested from two mice in each *Tlr4^flox/flox^* and *Tlr4^fl/fl^/LysM‐cre* group on day 7 post anti‐GBM cGN induction. Single cell suspension of entire renal tissue was generated by 30 min incubation of minced kidney tissue in 0.1 mg mL^−1^ of Blendzyme 4 (Roche Inc., Indianapolis, IN) at 37 °C on a rotating shaker (200 rpm). Supernatants of the digested samples were collected in RPMI with 10% FBS on ice, followed by meshing through a 40 µm strainer with the syringe head. The total digested renal single cells were centrifuged at 650 relative centrifugal force (rcf) at 4 °C for 5 min, followed by red blood cell lysis. For FACS of live CD45^+^ cells, digested and red blood lysed single cell suspension was stained with DAPI for nuclei (Invitrogen) and anti‐mouse CD45‐PE antibody (BD Biosciences) on ice, and single cells were sorted on a flexible BD Influx cell sorter (BD Biosciences) with 100 µm nozzle. Cell viability was assessed after sorting via Countess 77 II FL.

### Chrimium cDNA Library Preparation and scRNA‐seq

For each group, FACS sorted live CD45^+^DAPI^–^ cells from flexible BD Influx cell sorter (BD Biosciences) with qualified viability, were counted and loaded for 10× Chromium scRNA‐seq Library preparation and Illumina sequencing (Pair‐End sequencing of 151 bp) at the University of Hong Kong, LKS Faculty of Medicine, Centre for PanorOmic Sciences, Genomics Core. FACS sorted live CD45^+^ DAPI^−^ cells were loaded onto the 10× Chromium Single Cell chip for each group, and encapsulated into Gel in‐beads emulsions (GEM) by 10× Chromium Controller. Single Cell 5′ Reagent Kits was used to perform downstream steps. Illumina NovaSeq 6000 was used for Pair‐End 151 bp sequencing. Primarily, raw sequencing data were converted to fastq files with the Illumina bcl2fastq tool. Then, the 10× Genomics CellRanger pipeline was used to export gene expression matrix for each group, as well as an additional application of CellRanger module aggr function to aggregate libraries from *Tlr4^flox/flox^* and *Tlr4^fl/fl^/LysM‐cre* group for comparative analysis.

### Single Cell Mapping and Clustering

The UMI counts for each gene in each cell were imported into R (version 4.1.2) by Seurat package (version 4.0.5). Quality control was assessed priorly, by filtering out cells with the following criteria: less than 200 or more than 6000 unique genes expressed, or more than 6% of reads mapping to mitochondria, resulting in a final data set of 12 399 cells (6498 from *Tlr4^flox/flox^* mice and 5901 from *Tlr4^fl/fl^/LysM‐cre* mice). Sequencing data from each group was further normalized and scaled, and the detected for highly variable features by using Normalized function, ScaleData and FindVariableFeartures function, respectively, in Seurat package. Thereafter, principal component analysis (PCA) and linear dimensional reduction based on the selected top 3000 high variable genes on the scaled data were performed. The dimensionality was determined in accordance with the resampling test^[^ ^51^ ^]^ for the subsequent cell subgroup clustering. Libraries from *Tlr4^flox/flox^* and *Tlr4^fl/fl^/LysM‐cre* groups were pooled into one combined library by Cell Ranger aggr function for batch correction.^[^ ^52^ ^]^ To avoid insufficient or overclustering, Clustree R Bioconductor package (version 0.4.4) was used in advance, to help validate the legitimacy of resolution selection for cell clustering.^[^ ^53^ ^]^ Resolution at 0.65 was chosen for cell clustering for the integrated library (Figure S1, Supporting Information). Subsequently, cells were clustered into 19 groups for myeloid TLR4‐dependent cell type identification using FindNeighbors and FindClusters function in Seurat package.

### Differential Expression Analysis

Marker genes in each cluster were determined using Wilcoxon Rank Sum test via the FindAllMarkers function in Seurat, with a false discovery rate adjusted *p*‐value (FDR) cut‐off of 0.05. DEGs in individual cell type between different experimental conditions and between different cell clusters were recognized under limitation of an estimated log fold change >0.25 and detectable expression in >10% of cells in any of the compared cell cluster by FindMarkers function in Seurat. Relevant cell marker genes expression was visualized on UMAP plot and Violin Plot to illustrated specific cell genotypes (Figures S3 and S8, Supporting Information).

### Functional Enrichment Analysis

Macrophage functional‐related gene set enrichment was tested via GSEABase (version 1.56.0) and GSVA R package (version 1.42.0) within a panel of annotated gene database (Gene Ontology: http://software.broadinstitute.org/gsea/msigdb/genesets.jsp?collection=BP, Reactome: http://software.broadinstitute.org/gsea/msigdb/genesets.jsp?collection=CP:REACTOME, Kyoto Encyclopedia of Genes and Genomes: http://software.broadinstitute.org/gsea/msigdb/genesets.jsp?collection=CP:KEGG, Human Immunosuppression Gene Atlas: http://biokb.ncpsb.org/HisgAtlas). The enrichment score of gene sets at single‐cell level was calculated and visualized by AUCell (version 1.16.0) R Bioconductor package based on gene ranking based expression matrix of each cell. Unbiased gene regulatory network was constructed based on the shortest path algorithm interaction networks found between the cell type specific DEGs by Metacore analytical suite (version 4.2 build 8168; GeneGo, St Joseph, MI).^[^ ^27^ ^]^

### Single‐Cell Regulatory Network Inference and Clustering

To reveal the putative transcription factors and their direct downstream targets (regulon) activities in each macrophage subpopulation, RcisTarget Bioconductor and SCENIC algorithm for construction of gene networks in accordance of the developer's program pipeline were used.^[^ ^16^ ^]^ Motif TFs databases for mouse were loaded for motif enrichment calculation from the following links: https://resources.aertslab.org/cistarget/databases/mus_musculus/mm9/refseq_r45/mc9nr/gene_based/mm9-500bp-upstream7species.mc9nr.feather and https://resources.aertslab.org/cistarget/databases/mus_musculus/mm9/refseq_r45/mc9nr/gene_based/mm9-tss-centered-10kb-7species.mc9nr.feather. Gene expression matrixes of each sample were analyzed by GENIE3 package in the Bioconductor for identification of regulatory modules and potential TF targets within every inputted single cell. Activity of each regulon was scored in individuals by calculating the AUC using AUCell package in bioconductor. Then the score was ranked within each cell, followed by motif enrichment analysis, visualized in heatmap and UMAP plot.

### Cell Development Trajectories Construction

Single‐cell pseudotime trajectories of monocyte/ macrophage populations were reconstructed by Monocle 2 (version 2.22.0) package. Genes used for pseudotime ordering were taken from DEGs identified by function differentialGeneTest with fullModelFormulaStri set. Dimension reduction and cell ordering along the trajectories were completed with DDRTree method. Dynamic changes in classical (M1) and alternative (M2) macrophage associated genes^[^ ^54^ ^]^ expression with pseudotime trajectories were illustrated as heatmap.

### Flow Cytometry Analysis

Following systemic perfusion with cold PBS through left ventricle, kidneys were harvested for flow cytometry for both *Tlr4^flox/flox^* and *Tlr4^fl/fl^/LysM‐cre* mice on the seventh day post anti‐GBM cGN induction. Single cell suspension of entire renal tissue was prepared as previously described.^[^ ^11^ ^]^ Cells were then Fc‐blocked (BD Biosciences, San Jose, CA) for 30 min on ice with 10% FBS in PBS after red blood cell lysis. Afterward, cells were stained in dark with the pre‐conjugated antibody cocktails for 60 min on ice. For the intracellular staining, such as NUR77, cells were fixed with IC fixation buffer and permeabilized with Permeabilization Buffer (eBioscience) before staining. Cells with or without irrelevant antibody staining were used as negative controls. The antibodies used in the study were listed in **Table** **1**. Flow cytometry was performed on a BD LSRFortessa^TM^ using the BD FACSDiva software, and analyzed by FlowJo software (version 7.6.1) for quantitative analysis.

**Table 1:** List of antibodies used in flow cytometry and immunofluorescent staining

Target	Concentration	Conjugate	Catalog number	Manufacturer	Clone
CD11b	1:100	Alex Flour 488	53‐0112‐82	eBioscience	M1/70
CD68	1:100	BV421	137017	Biolegend	FA‐11
F4/80	1:100	APC	123116	Biolegend	BM8
F4/80	1:100	Unconjugated	MCA497GA	Serotec	Cl:A3‐1
Nr4a1	1:100	PE	12‐5965‐82	eBioscience	12.14
RNASE2	1:100	Unconjugated	PA5‐60882	Invitrogen	Polyclone
Rat IgG	1:200	Peroxidase	P0450	Dako	
Rabbit IgG	1:200	Polymer‐HRP	K4003	Dako	

### Opal Multiplex Immunofluorescence

Formalin‐fixed paraffin mouse tissue (kidney and spleen) sections (3 µm thickness) were used for opal multiplex immunofluorescence staining. After deparaffinization, rehydration, 30‐min endogenous horseradish peroxidase (HRP) blocking (3% hydrogen peroxide solution) and microwave‐based antigen retrieval in citrate buffer in turn, sections were incubated with primary antibodies, including F4/80, RNASE2 (EAR2) as listed in Table 1 at 4 °C overnight in a humidified chamber. On the next day, slices were washed in TBST 10 min for three times, and then incubated with appropriate HRP‐conjugated secondary antibodies for 1 h at room temperature. The fluorescence was developed using the Alex Flour 488 Tyramide Reagent (B40955, Invitrogen, Carlsbad, CA) and Alex Fluor 568 Tyramide Reagent (B40956, Invitrogen) according to the manufacturer's instruction, followed by nuclei counterstaining with Hoechst 33342, Trihydrochloride, Tridydrate (H1399, Invitrogen).

### Cell Culture

Bone marrow was flushed from tibia and femur of genotype‐validated *Tlr4^flox/flox^* and *Tlr4^fl/fl^/LysM‐cre* mice with C57BL/6 background, for preparation and development of BMDMs following a well‐established protocol.^[^ ^38^, ^55^ ^]^ BMDMs were differentiated by 50 ng mL^−1^ M‐CSF in RPMI 1640 medium (21875034, Gibco) containing 10% FBS and 100 U mL^−1^ penicillin and streptomycin (P/S) (Life Technologies) at 5% CO_2_ humidified incubator at 37 °C for 7 d. The culture medium with M‐CSF was replaced once every 3 d.

### Small Interfering RNA Transfection

*Nr4a1* and *Ear2* were knocked down on BMDMs from *Tlr4^flox/flox^* and *Tlr4^fl/fl^/LysM‐cre* mice, by transfecting small interfering RNA (siRNA) specific for *Nr4a1* (5′‐CGGCGUCCUUCAAGUUUGATT‐3′), *Ear2* (5′‐CAUCCAGCAUAUCAAUAAU‐dTdT‐3′), and nonsense control (5′‐UUCUCCGAACGUGUCACGUTT‐3′) for 12 h, respectively, with Lipofectamine RNAiMAX transfection system (Life Technologies, NY, USA) and Opti‐MEM medium (Gibco, Thermo Fisher Scientific, MA, USA). Transfected BMDMs were collected on 3‐ and 6‐h post LPS stimulation (500 ng mL^−1^) for real‐time PCR analysis and 48 h after LPS stimulation (500 ng mL^−1^) for ELISA analysis.

### Quantitative Real‐Time PCR Assay

Total mRNAs from kidney tissue or culture cells were extracted and purified using the RNeasy Kit (Qiagen, Düsseldorf, Germany) following the manufacturer's instructions. Promega reverse transcription system was employed for complementary DNA (cDNA) synthesis. Quantitative real‐time PCR was performed on QuantStudio 7 Flex Real‐time PCR platform using SYBR Green Supermix (Bio‐Red). Primers used in this study are listed in **Table** **2**. The ratio of tested mRNA was normalized to GAPDH mRNA expression, as the internal control, by the formular: ΔCt = 2^ [Ct (target gene) − Ct (GAPDH)].

**Table 2:** List of primers used in real‐time PCR

Gene	Species	Forward primer	Reverse primer
*Cd36*	Mouse	CTCCTAGTAGGCGTGGGTCT	TGGCTTCAGGGAGACTGTTG
*Ear2*	Mouse	CACAAAGCAGACAGGGAAACAT	TCTTCTAAACCTGTTAATACGCTGC
*GAPDH*	Mouse	TGCTGAGTATGTCGTGGAGTCTA	AGTGGGAGTTGCTGTTGAAATC
*Ifn*	Mouse	TTTCGCCTTGCTGTTGCTGA	TGGATATCTGGAGGAACTGGCA
*Il1b*	Mouse	CTTCAGGCAGGCAGTATCACTCAT	TCTAATGGGAACGTCACACACCAG
*Ccl2*	Mouse	CTTCTGGGCCTGCTGTTCA	CCAGCCTACTCATTGGGATCA
*Nr4a1*	Mouse	TTGAGTTCGGCAAGCCTACC	GTGTACCCGTCCATGAAGGTG
*Rela*	Mouse	GTGCCTCCCAGCCTGGT	AGATCTCTTCTTGCTGTGCGA
*S100a8*	Mouse	ACTTCGAGGAGTTCCTTGCG	TGCTACTCCTTGTGGCTGTC
*Tgfβ1*	Mouse	CAGTGGCTGAACCAAGGAGAC	ATCCCGTTGATTTCCACGTG
*Tlr4*	Mouse	CATCCAGGAAGGCTTCCACA	GGCGATACAATTCCACCTGC
*Tnf*	Mouse	CATCTTCTCAAAATTCGAGTGACAA	TGGGAGTAGACAAGGTACAACCC

### ELISA

Levels of EAR2 in kidney homogenate extract samples were measured by using ELISA kits (mbs2611440, MyBioSource, CA, USA) following the manufacturer's instructions. Freshly prepared ABTS working solution was added to each sample for initiation of the reactions in the dark. The optical density (OD) was determined by microplate reader at 450 nm. The final levels of EAR2 were normalized to ng per mL.

### ChIP Assay

It was found that mRNA expression of *Nr4a1*, encoding a rapid response protein NUR77 in macrophages, was peaked 1 h after LPS stimulation in BMDMs (Figure 6A). Whereas, NUR77 expression at the protein level is reported as early as 1 h, peaking over 6–12 h post LPS stimulation in RAW 264.7.^[^ ^35^ ^]^ In addition, the *Ear2* mRNA expression continuously accelerated in *Tlr4^fl/fl^/LysM‐cre* BMDMs from 3 to 6 h after LPS stimulation (Figure 6A). Therefore, 6‐h LPS stimulation was selected as the optimal time point for ChIP assay. The BMDMs from *Tlr4^flox/flox^* and *Tlr4^fl/fl^/LysM‐cre* mice, with or without 12‐h siRNA transfection for *Nr4a1* knockdown or nonsense control, were treated with 500 ng mL^−1^ LPS for 6 h. Cells were then collected for ChIP Assay by using SimpleCHIP Enzymatic Chromatin IP Kit (Magnetic Beads) (#9003, Cell Signaling, Danvers, MA) according to the manufacturer's instruction. Briefly, immunoprecipitation was conducted with anti‐mouse NUR77(Nr4a1) (1:100) (sc‐365113X, Santa Cruz, TX). IgG was used as a negative control (#3900, Cell Signaling). Precipitated DNAs were identified by PCR. Primers targeting the predicted NUR77 biding site on the conserved region of mouse *Ear2* 3′ UTR were use with sequence: forward (5′‐GGCTACAGACTGACACCCTA ‐3′) and reverse (5′‐CCCTGGGATCCTCTTGCAAT ‐3′).

### Statistical Analysis

Statistical tests were performed using Prism 5.0 GraphPad Software (GraphPad Software, La Jolla, CA). Data obtained from this study were expressed as the mean ± SEM. Two‐group comparisons were performed using an independent sample *t*‐test unless otherwise indicated. Multiple group comparisons were performed using one‐way analysis of variance (ANOVA) followed by Tukey's post‐hoc tests. Differences with a *p‐*value less than 0.05 were considered statistically significant.

## Conflict of Interest

The authors declare no conflict of interest.

## Supporting information

Supporting Information

## Data Availability

The data that support the findings of this study are available from the corresponding author upon reasonable request.
