# Correction to “Single‐Cell RNA Sequencing Identified Novel Nr4a1^+^ Ear2^+^ Anti‐Inflammatory Macrophage Phenotype under Myeloid‐TLR4 Dependent Regulation in Anti‐Glomerular Basement Membrane (GBM) Crescentic Glomerulonephritis (cGN)”

**DOI:** 10.1002/advs.76055

**Published:** 2026-06-12

J Chen, XR Huang, F Yang, WH Yiu, X Yu, SCW Tang, HY Lan, “Single‐cell RNA Sequencing Identified Novel Nr4a1^+^ Ear2^+^ Anti‐Inflammatory Macrophage Phenotype under Myeloid‐TLR4 Dependent Regulation in Anti‐Glomerular Basement Membrane (GBM) Crescentic Glomerulonephritis (cGN),” *Advanced Science* 9, no. 18 (2022): e2200668. https://doi.org/10.1002/advs.202200668

In Figure 5D, the authors admitted to an error in which one spleen image from a *Tlr4^f/f/LysM‐cre^* normal control mouse was mistakenly placed in the panel representing a *Tlr4^flox/flox^* normal control mouse during final figure assembly. The incorrect and corrected images originated from different imaging sessions and batches, confirming that the error occurred only during the arrangement of representative panels when assembling the composite figure, not during staining, imaging, or file export. The incorrect image has now been replaced with the correct *Tlr4^flox/flox^* normal control mouse spleen image from a separate experiment batch, an independent staining and imaging session. In addition, the authors subsequently re‐verified the source files for all figures in the manuscript to ensure full consistency between raw data and published figures. The authors emphasize that this error involved only a representative immunofluorescence image from normal mouse spleen tissue. It does not affect any images from the anti‐GBM disease groups, any experimental comparisons, or any of the quantitative results in this study, which were independently performed using flow cytometry data. Therefore, this correction does not affect the results, data interpretation, or conclusions of the study. The corrected Figure 5D is shown as follows.

The corrected Figure 5D is shown in the next page.


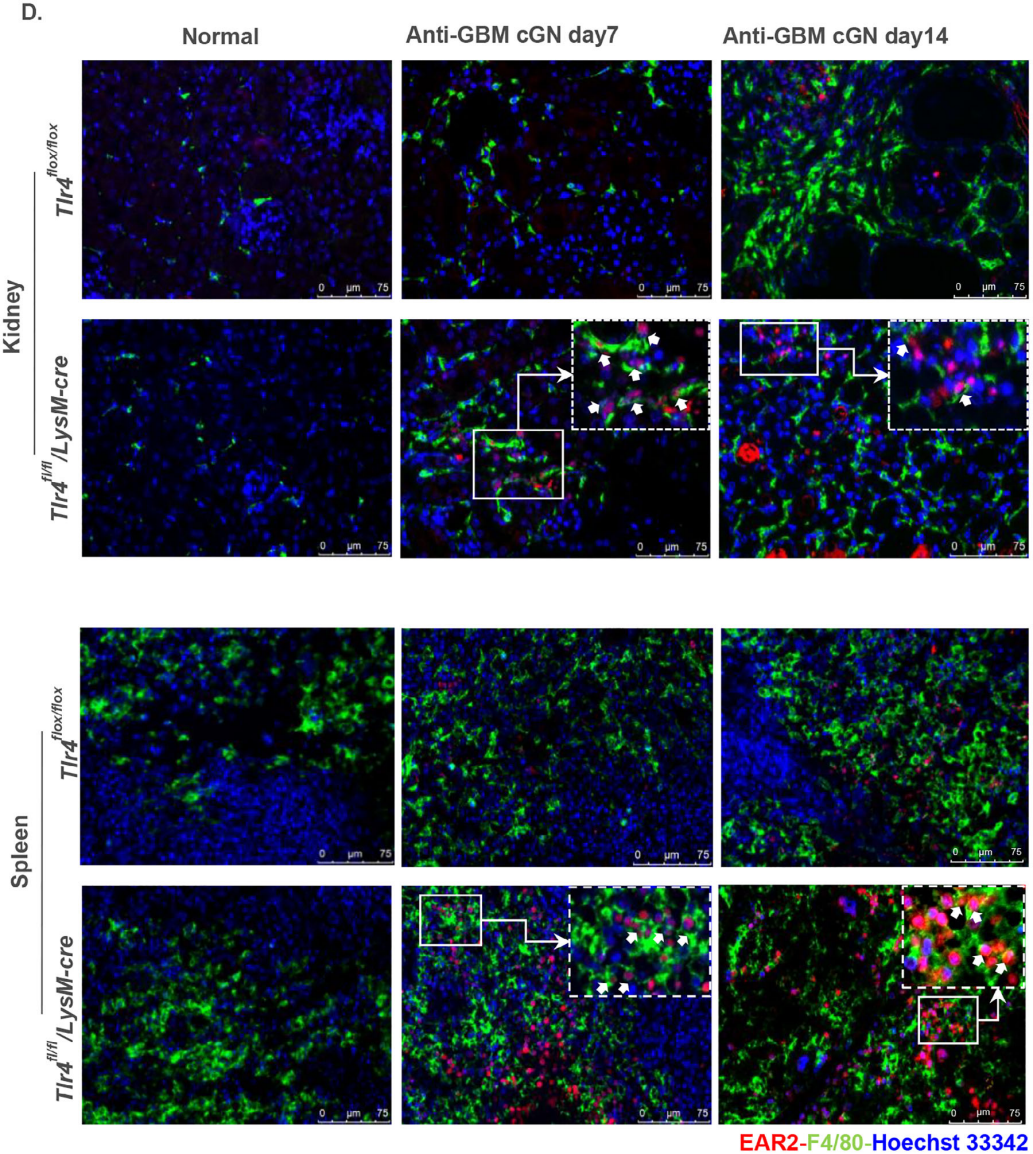


The authors apologize for this error.

