# Supplementary material for: Single‐cell RNA Sequencing Identified Novel Nr4a1+ Ear2+ Anti‐Inflammatory Macrophage Phenotype under Myeloid‐TLR4 Dependent Regulation in Anti‐Glomerular Basement Membrane (GBM) Crescentic Glomerulonephritis (cGN)
Source: Adv Sci (Weinh). 2022 Apr 28;9(18):2200668. doi: 10.1002/advs.202200668 (PMC9218767; doi:10.1002/advs.202200668)
Supplement: Supplementary file 1 — Supporting Information [file ADVS-9-2200668-s001.pdf]

ADVANCED SCIENCE
Open Access

Supporting Information

for *Adv. Sci.*, DOI 10.1002/advs.202200668

Single-cell RNA Sequencing Identified Novel Nr4a1^{+} Ear2^{+} Anti-Inflammatory Macrophage Phenotype under Myeloid-
TLR4 Dependent Regulation in Anti-Glomerular Basement Membrane (GBM) Crescentic Glomerulonephritis (cGN)

*Jiaoyi Chen, Xiao Ru Huang, Fuye Yang, Wai Han Yiu, Xueqing Yu, Sydney C. W. Tang and Hui Yao Lan**

Single-cell RNA Sequencing Identified Novel Nr4a1^{+} Ear2^{+} Anti-inflammatory Macrophage Phenotype under Myeloid-TLR4 Dependent Regulation in anti-Glomerular Basement Membrane (GBM) crescentic glomerulonephritis (cGN)

*Jiao-Yi Chen, Xiao-Ru Huang, Fu-Ye Yang, Wai-Han Yiu, Xue-Qing Yu, Sydney Chi-Wai Tang, Hui-Yao Lan**

Supporting information

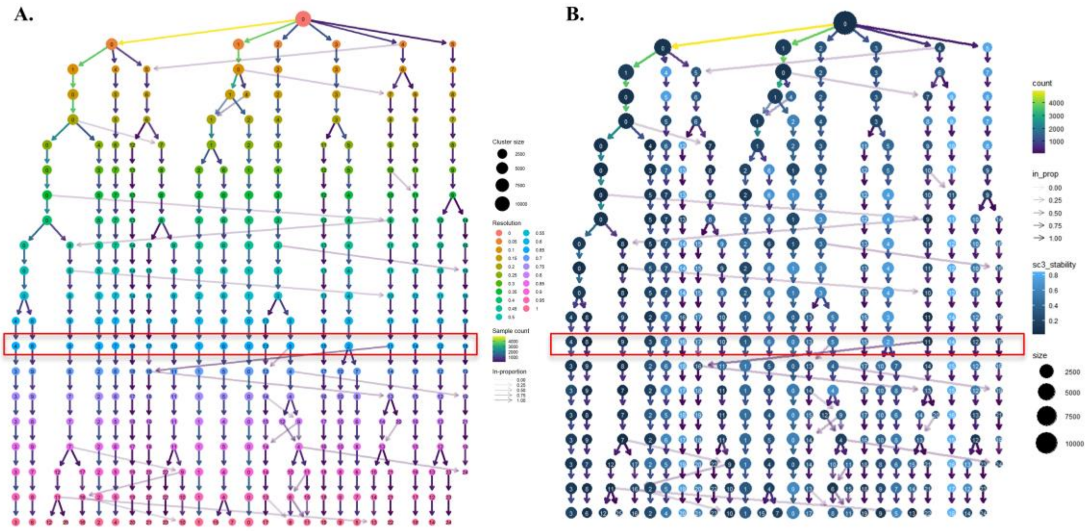

Figure S1. Clustering tree of resolution parameters calculated via Seurat package, ranging from zero to 1.0. A) Default clustering tree of resolution parameters. At the highest resolutions, we begin to see many low in-proportion edges, indicating cluster instability. Seurat labels clusters according to their size, with cluster 0 involving the largest cell population. B) Clustering tree with nodes colored by the SC3 stability index from light-blue (lowest) to dark-blue (highest) are shown. Red frame indicates resolution 0.65, used in this study.

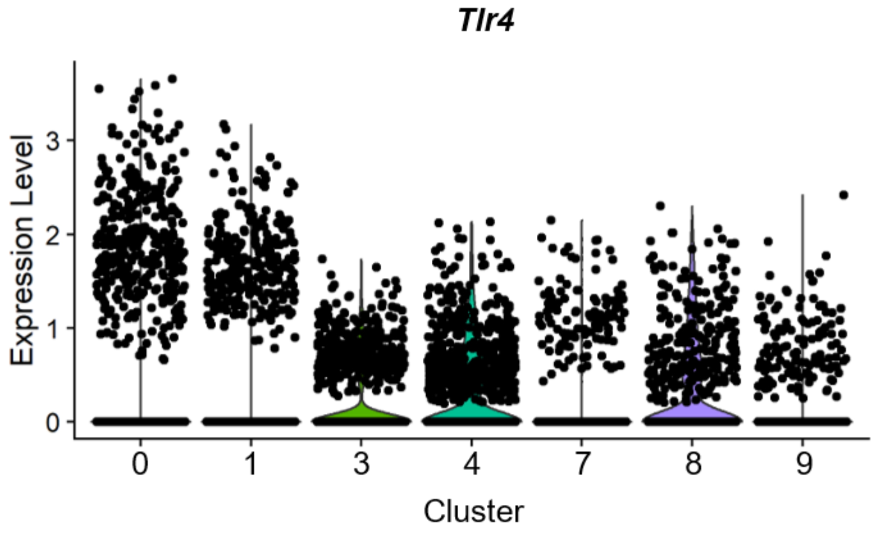

Figure S2. Vlnplot of *Tlr4* expression level identified within monocyte/macrophage populations in scRNA-seq profile.

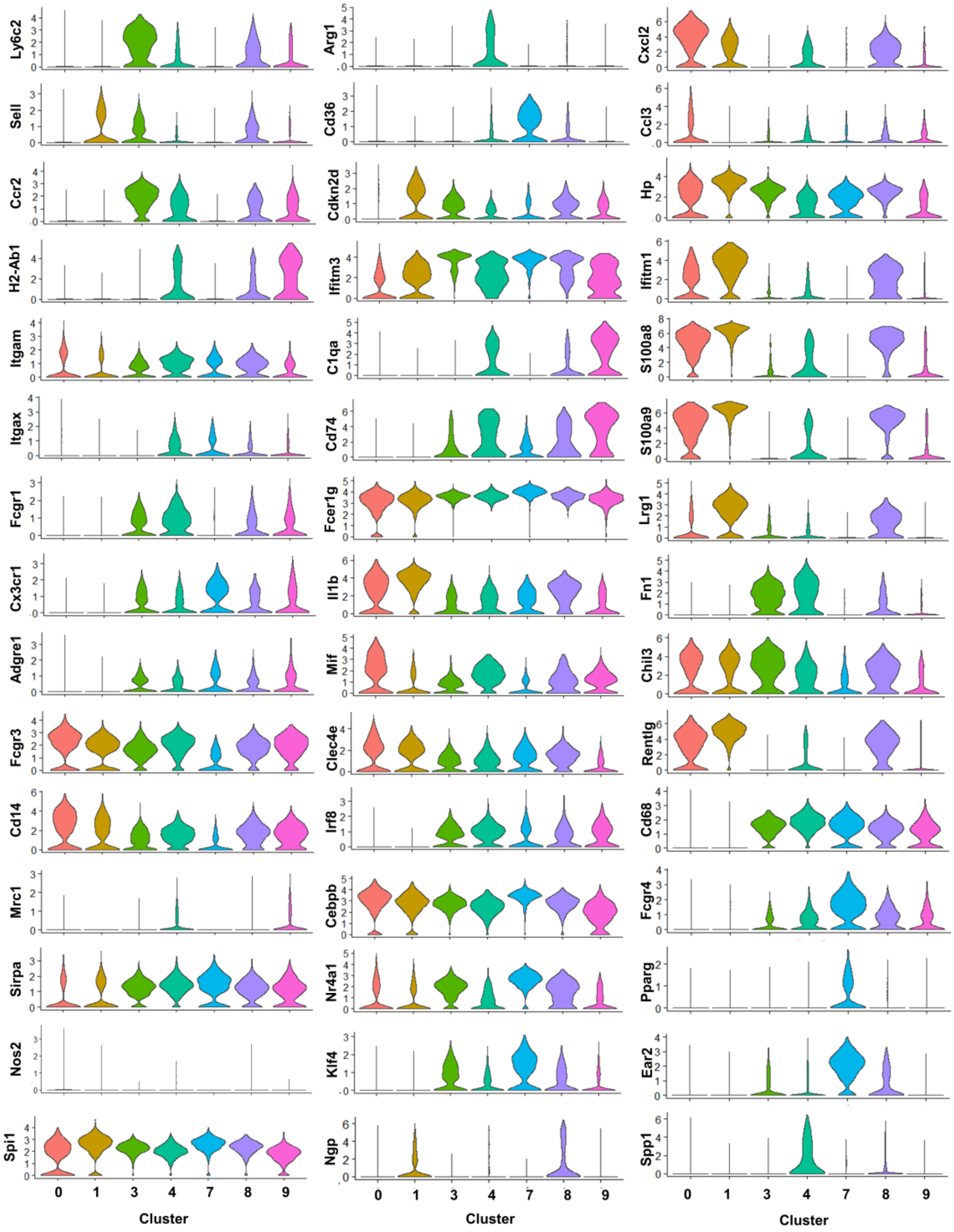

Figure S3. Vlnplots of gene expression level identified within the monocyte/macrophage populations.

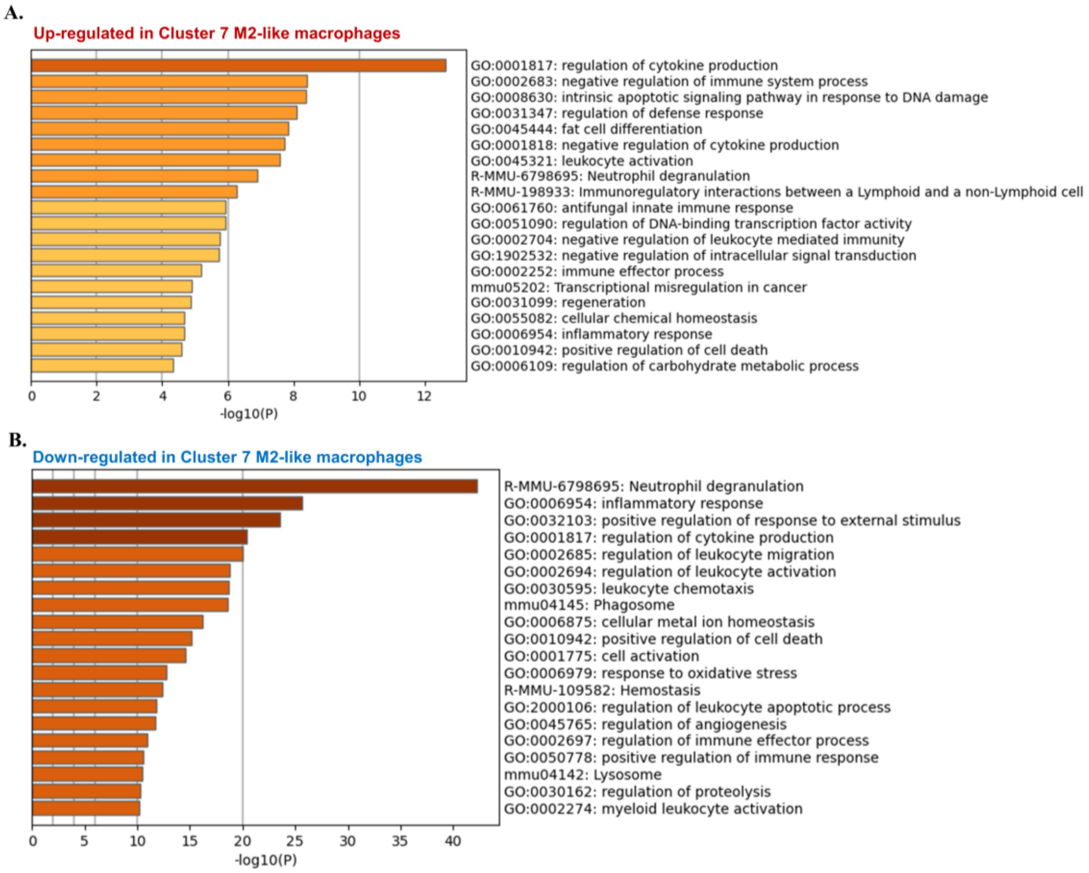

Figure S4. Enrichment analysis of Cluster 7 DEGs compared to Cluster 3 and 4 M2 macrophages. Barplots show the enrichment analysis for the up-regulated 103 DEGs (A) and the down-regulated 378 DEGs (B) in Cluster 7 macrophages, compared to Cluster 3 and Cluster 4 M2 macrophages**.**

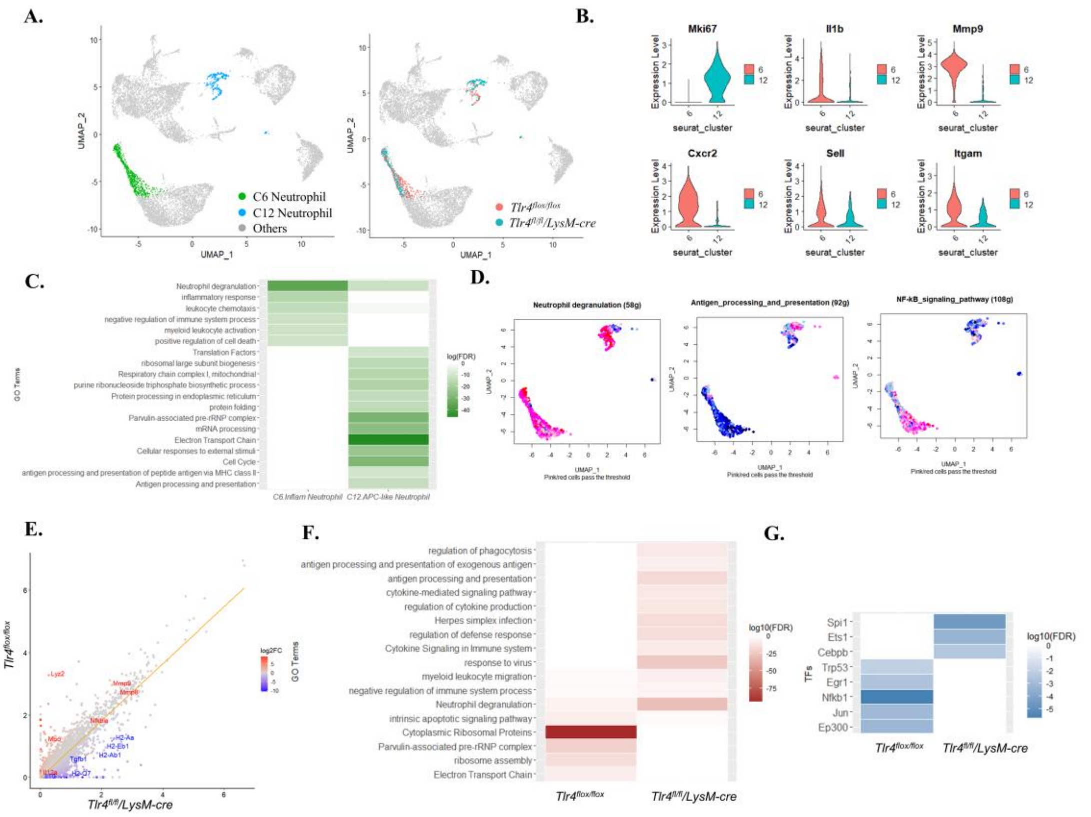

Figure S5. Deletion of myeloid-TLR4 suppresses proinflammatory neutrophil infiltration and activation. A) UMAP visualization of two transcriptionally distinct kidney neutrophil subpopulations (Cluster 6, and 12) in *Tlr4^flox/flox^* and *Tlr4^fl/fl^/LysM-cre* mice at day 7 post anti-GBM cGN. Each point depicts a single cell, colored according to cluster designation (left plot) or genotypes (right plot). B) Violin plot illustrating the normalized expression level of *Mki67, Il1b, Mmp9, Cxcr2, Sell (CD62L), Itgam (CD11b)* in Cluster 6 and Cluster12 neutrophils. C) Heatmap shows top significantly enriched terms across up-regulated DEGs in Cluster 6 inflammatory neutrophils and Cluster 12 antigen presenting cells-like neutrophils, coloured by log10(FDR). D) Colored UMAP plots demonstrating neutrophils with activated gene sets related to neutrophil degranulation [Reactome: R-MMU-R-MMU-6798695], antigen processing and presentation [KEGG: mmu04612], and NF-κB signaling

pathway [KEGG: mmu04064] based on AUC scores. Each point depicts a single cell. Cells that pass the assignment threshold of AUC enrichment scores are colored in shades of pink-red, and the cells that don't pass the threshold are colored in black-blue. E) Scatter plot of average expression of overall detected genes in both of *Tlr4$^{flox/flox}$ and Tlr4$^{fl/fl}$/LysM-cre* neutrophils, highlighting genes that exhibit TLR4-dependent dramatic responses under anti-GBM cGN condition. Each dot represents a gene, colored in accordance with the average log2 (Foldchange) (log (FC)), with scale bar on the right. F-G). Heatmap shows the top significantly enriched terms (F) and transcriptional regulatory interaction (G) across up-regulated DEGs in Cluster 6 proinflammatory neutrophils from *Tlr4$^{flox/flox}$ and Tlr4$^{fl/fl}$/LysM-cre* group respectively, coloured by log10(FDR). All the enrichment analysis has been carried out with the following ontology sources: KEGG Pathway, GO Biological Processes, Reactome Gene Sets, CORUM, TRRUST and PaGenBase. DEGs, differentially expressed genes; FDR, false discovery rate.

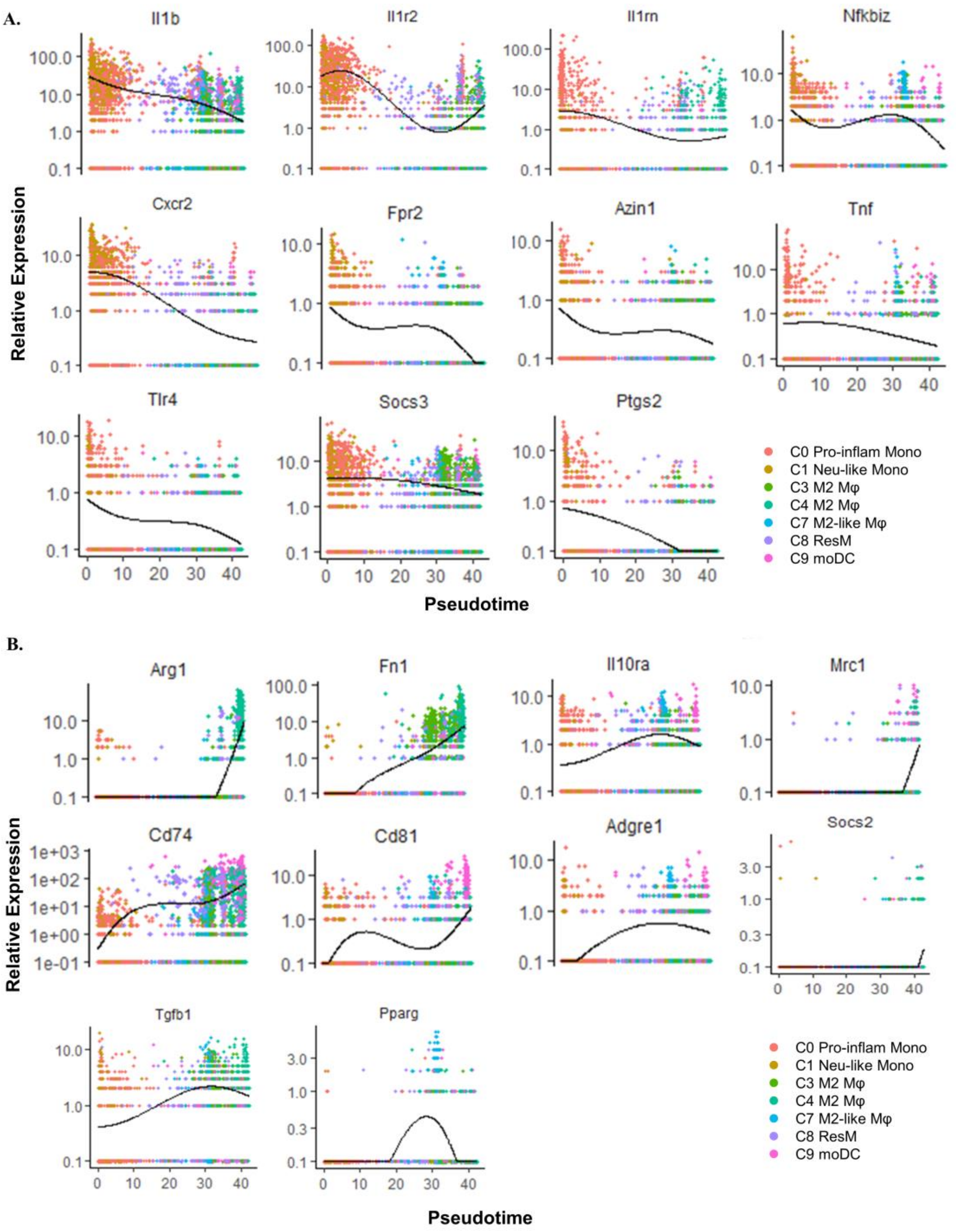

Figure S6. Expression pattern of representative M1 and M2 markers alongside the pseudotime trajectory. Dot plots illustrates the expression patterns of A) M1 and B) M2 macrophage marker genes alongside the pseudotime. Each dot represents one cell. Cells are colored according to cluster designation. Pro-inflam Mono: pro-inflammatory monocyte, Neu-like Mono: neutrophil-like monocyte, Mφ: macrophage, ResM: resident macrophage, moDC: monocyte-derived dendritic cell.

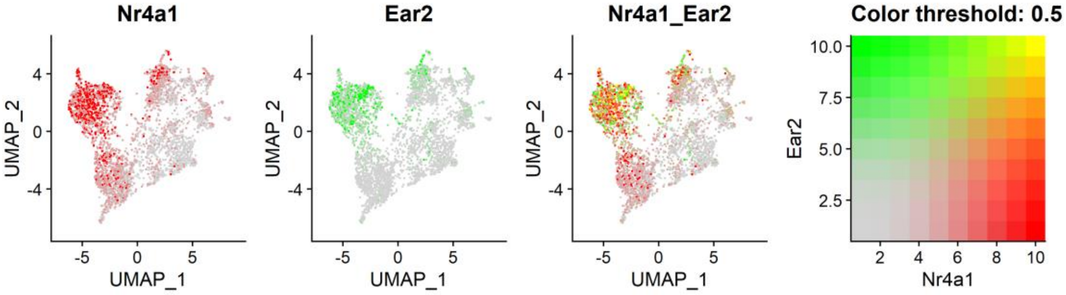

Figure S7. Visualization of co-expression of *Nr4a1* and *Ear2* among macrophage subsets. A) UMAP plots shows re-clustered cell distribution of Cluster 3, 4, 7, 8 macrophages subsets. Cells with positive *Nr4a1* expression were colored red, with positive *Ear2* expression colored green. Cells in yellow demonstrate cells co-expressing *Nr4a1* and *Ear2*. B) Color threshold for (A).

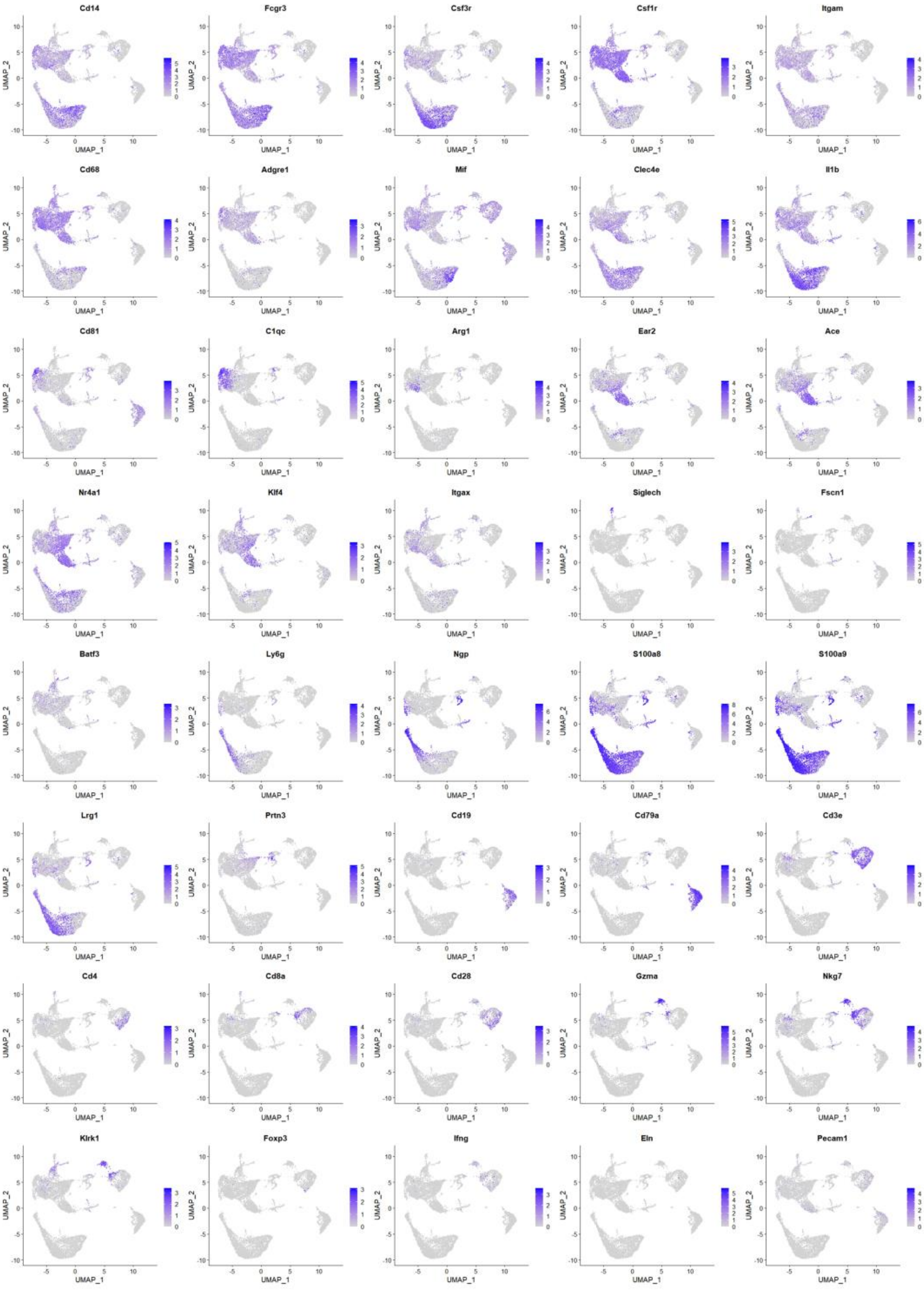

Figure S8. UMAP plots of gene expression gradients identified within cell populations. Each point depicts a single cell, colored according to normalized expression levels. Average expression scale is shown on the right side.

Table S1. List of DEGs in Cluster 7 macrophages compared to Cluster 3 and 4 macrophages

Gene	p_val	avg_log2FC	p_val_adj
Ace	3.22E-245	2.348521193	9.67E-242
Ear2	7.80E-191	2.082345066	2.34E-187
Id2	8.97E-155	1.858350033	2.69E-151
Nr4a1	2.45E-232	1.81192635	7.35E-229
Pglyrp1	1.02E-155	1.771978586	3.07E-152
Fcgr4	5.40E-113	1.738802718	1.62E-109
Ddit4	1.81E-130	1.629179926	5.42E-127
Cd300e	2.12E-119	1.61091082	6.35E-116
Gngt2	1.74E-253	1.513364634	5.21E-250
Dusp2	7.59E-135	1.510243444	2.28E-131
Dusp16	1.51E-58	1.459424651	4.52E-55
Cd36	6.25E-51	1.420784082	1.87E-47
Ccnd2	3.67E-209	1.376054896	1.10E-205
Ap1s2	8.88E-110	1.352142258	2.66E-106
Naga	1.96E-68	1.309280207	5.89E-65
Slc12a2	1.81E-74	1.222116832	5.42E-71
Grk3	2.69E-61	1.216744304	8.07E-58
Treml4	3.26E-125	1.185303985	9.77E-122
Pou2f2	2.25E-149	1.16390614	6.75E-146
Cst3	2.18E-187	1.141168459	6.54E-184
Cd300ld3	9.14E-36	1.136533538	2.74E-32
Dusp5	5.06E-28	1.045466092	1.52E-24
Fabp4	7.07E-28	1.025497759	2.12E-24
Smpdl3b	4.39E-31	1.018358108	1.32E-27
Cebpb	3.26E-178	1.008935912	9.78E-175
Spn	1.56E-94	0.996950022	4.68E-91
Gm15987	1.96E-31	0.975474793	5.87E-28
Fam46a	1.75E-33	0.964012899	5.24E-30
Mdm1	1.53E-07	0.917448032	0.000457546
Sh2d1b1	2.62E-17	0.869579479	7.86E-14
Smpdl3a	1.27E-77	0.863948083	3.82E-74
Lst1	5.26E-84	0.808440298	1.58E-80
Plac8	6.31E-41	0.804987393	1.89E-37
Klf4	6.08E-24	0.802464836	1.82E-20

Adgre4	5.19E-78	0.782790402	1.56E-74
Hfe	1.58E-18	0.775091147	4.74E-15
Nxpe4	2.79E-13	0.77023527	8.38E-10
Slc11a1	1.78E-42	0.752253297	5.33E-39
Fpr2	3.46E-107	0.74794666	1.04E-103
Gm36161	6.18E-52	0.742980956	1.85E-48
Cd274	3.83E-39	0.742723038	1.15E-35
Metrnl	3.28E-26	0.717221293	9.83E-23
Atp1a1	1.69E-61	0.706184457	5.08E-58
Ceacam1	1.78E-13	0.696869569	5.33E-10
Fcgrt	6.85E-11	0.692968802	2.05E-07
Sat1	1.52E-60	0.675769956	4.56E-57
Cd9	2.00E-21	0.669978418	5.99E-18
Hpgd	2.14E-27	0.661019691	6.43E-24
Clec4e	3.18E-14	0.653796436	9.53E-11
Serpinb6a	1.93E-31	0.625305467	5.80E-28
Rbpms	1.16E-19	0.623054106	3.49E-16
Phlda3	1.75E-124	0.61817094	5.25E-121
Clec4a1	9.93E-42	0.614708979	2.98E-38
Gpx1	2.69E-103	0.608616885	8.08E-100
Bcl2a1d	3.49E-19	0.583224352	1.05E-15
Gm21188	1.40E-31	0.571018197	4.19E-28
AW112010	2.28E-38	0.565111643	6.84E-35
Rnase6	5.38E-12	0.550940864	1.61E-08
Cx3cr1	2.59E-12	0.549320555	7.76E-09
Eno3	2.01E-33	0.549019696	6.02E-30
Clec4a3	9.39E-35	0.544153525	2.82E-31
Hes1	2.86E-13	0.535636734	8.59E-10
Cd300a	3.02E-45	0.533080519	9.07E-42
Hsd11b1	1.77E-06	0.517461306	0.005300428
Nupr1	7.32E-27	0.510876946	2.20E-23
Cybb	2.06E-36	0.503725545	6.19E-33
H2-Q7	9.35E-06	0.465643783	0.028054302
Bcl2a1a	4.45E-24	0.464637463	1.33E-20
Cd300c2	2.54E-31	0.459259766	7.61E-28
Lilra5	2.21E-13	0.458620699	6.63E-10
Pla2g7	2.89E-14	0.457958688	8.68E-11
Rnaseh2b	7.37E-74	0.445363808	2.21E-70

Heg1	8.14E-08	0.440197196	0.000244078
Nucks1	1.16E-77	0.422286251	3.48E-74
Gm42418	4.52E-17	0.40908944	1.36E-13
Nfkbid	3.27E-13	0.403327695	9.80E-10
Id3	1.50E-06	0.397492718	0.004490961
Gm6377	3.13E-61	0.392639657	9.38E-58
Htra3	4.37E-153	0.390649607	1.31E-149
Dpep2	1.02E-10	0.383798777	3.06E-07
Bcl2	1.18E-18	0.379294692	3.54E-15
Rcan1	1.12E-18	0.367516831	3.35E-15
Clec2i	2.27E-08	0.366358936	6.80E-05
Cks2	2.77E-09	0.361945879	8.30E-06
Serpinb2	4.18E-08	0.354543556	0.000125396
Chka	8.09E-75	0.351936812	2.43E-71
Tbc1d8	5.62E-33	0.343700368	1.69E-29
Tcf7l2	9.10E-41	0.337465133	2.73E-37
Zbtb20	8.34E-80	0.334725917	2.50E-76
Trem3	9.16E-10	0.32035511	2.75E-06
Nectin1	4.04E-12	0.318528026	1.21E-08
Cd83	1.30E-50	0.314353004	3.91E-47
Ets2	3.40E-20	0.31430459	1.02E-16
Pmaip1	7.53E-08	0.312687957	0.000225844
Gpr65	1.40E-26	0.307292579	4.20E-23
Pld4	1.19E-13	0.298738433	3.56E-10
Wdfy4	1.44E-20	0.288774755	4.31E-17
Cd81	3.63E-98	0.285362187	1.09E-94
Sptbn1	1.12E-06	0.279893004	0.003373814
Hjurp	3.18E-70	0.268230485	9.53E-67
Bhlhe40	1.54E-05	0.260771427	0.046174917
Traf1	8.43E-14	0.258475191	2.53E-10
Ikbke	1.11E-29	0.252214039	3.34E-26
Pdlim4	5.85E-130	-0.25031763	1.75E-126
Serpinb8	2.03E-69	-0.252353379	6.09E-66
Slfn4	2.11E-25	-0.252447266	6.33E-22
Tubb6	2.22E-58	-0.25291027	6.66E-55
Basp1	3.14E-25	-0.253421541	9.42E-22
Il7r	1.68E-49	-0.256480799	5.03E-46
Snrpa	4.27E-24	-0.257082757	1.28E-20

Actn1	9.76E-56	-0.257697915	2.93E-52
Nme1	2.62E-25	-0.258097389	7.87E-22
Fh1	1.36E-90	-0.258407496	4.07E-87
Apex1	1.62E-163	-0.260709686	4.85E-160
Gusb	1.31E-36	-0.260969196	3.92E-33
Plscr1	1.61E-215	-0.261464256	4.84E-212
Ero1l	2.21E-136	-0.263115047	6.63E-133
Slc48a1	6.28E-85	-0.26354349	1.88E-81
Nrp2	0	-0.264711908	0
Sgk1	8.36E-29	-0.26556371	2.51E-25
Plbd1	2.51E-14	-0.265988977	7.53E-11
Creb5	2.51E-61	-0.266227172	7.53E-58
Arg2	3.06E-136	-0.266689494	9.18E-133
Ahcy	1.46E-179	-0.26715864	4.37E-176
Blvrb	8.03E-42	-0.267249589	2.41E-38
Bst2	3.51E-40	-0.268883333	1.05E-36
Snx24	1.16E-89	-0.269732178	3.49E-86
Stfa2	3.96E-06	-0.269740356	0.011873946
2510009E07Rik	9.79E-183	-0.270885948	2.94E-179
Tns3	1.55E-94	-0.271347082	4.64E-91
Lrrc59	2.66E-157	-0.271706732	7.97E-154
Mrps28	4.22E-96	-0.271772229	1.27E-92
Itga5	2.60E-85	-0.276489078	7.81E-82
1700017B05Rik	4.80E-72	-0.279587934	1.44E-68
Sigmar1	1.12E-117	-0.280332356	3.35E-114
Lsr	3.11E-50	-0.280373896	9.32E-47
Ccl3	2.15E-95	-0.281299535	6.45E-92
Dctpp1	3.58E-185	-0.283710571	1.08E-181
Abcc3	1.84E-125	-0.283740234	5.53E-122
Cxcr2	9.58E-13	-0.284820712	2.88E-09
Pmepa1	1.50E-97	-0.285325676	4.50E-94
Kcnn4	8.72E-120	-0.286792612	2.61E-116
2010005H15Rik	9.59E-100	-0.288272493	2.88E-96
Hacd4	2.98E-69	-0.289158534	8.95E-66
Axl	2.66E-49	-0.289340903	7.98E-46
Gm45716	3.24E-146	-0.290651758	9.71E-143
Phlda1	4.52E-71	-0.292103496	1.36E-67
Prnp	7.19E-48	-0.293169176	2.16E-44

AI839979	1.11E-06	-0.293660836	0.003327657
Txndc5	2.63E-98	-0.295230361	7.90E-95
Nsd2	1.34E-242	-0.295542057	4.03E-239
Snrpd1	5.93E-55	-0.296021916	1.78E-51
Dhrs7	1.05E-25	-0.296175002	3.16E-22
Ran	2.38E-30	-0.296382529	7.14E-27
Tnfrsf11a	7.00E-174	-0.297467624	2.10E-170
Kctd12	5.35E-84	-0.298630857	1.60E-80
Cebpa	2.18E-06	-0.298808685	0.006553853
Acot7	1.20E-134	-0.298895396	3.61E-131
Ldhb	6.46E-183	-0.300714652	1.94E-179
Hspd1	1.27E-103	-0.303888794	3.80E-100
Slc29a3	2.03E-103	-0.304292177	6.10E-100
Dhrs3	1.48E-72	-0.305251508	4.44E-69
Tarm1	1.36E-05	-0.30574747	0.040699496
Cd200r1	1.13E-99	-0.30770237	3.39E-96
Lmnb1	8.30E-121	-0.308229567	2.49E-117
Nenf	4.66E-33	-0.309765508	1.40E-29
Fnip2	2.40E-201	-0.309946176	7.21E-198
Nek6	3.26E-56	-0.31055385	9.77E-53
Adap2	1.89E-159	-0.310781099	5.67E-156
Isg15	2.36E-46	-0.312497173	7.08E-43
Ckap4	3.40E-98	-0.312791715	1.02E-94
H1f0	2.88E-06	-0.313352388	0.008643444
Tkt	8.17E-25	-0.313682283	2.45E-21
Pepd	1.46E-103	-0.314408131	4.37E-100
Sash1	3.60E-177	-0.316711871	1.08E-173
Rpn1	1.99E-30	-0.316939297	5.96E-27
P2rx7	4.04E-38	-0.317729868	1.21E-34
Renbp	2.26E-155	-0.318906969	6.78E-152
Tspan4	1.12E-56	-0.320863958	3.36E-53
Plk3	1.02E-149	-0.324528282	3.06E-146
Mmp19	2.33E-147	-0.326638273	7.00E-144
Plod1	1.12E-151	-0.327347588	3.35E-148
Atox1	9.42E-30	-0.329216267	2.82E-26
Txnrd1	1.74E-133	-0.331057588	5.23E-130
Hnrnpa3	4.64E-25	-0.336185337	1.39E-21
Atp6v1a	1.67E-59	-0.336383712	5.01E-56

Hebp1	9.02E-108	-0.336505778	2.71E-104
Dstn	1.63E-41	-0.337462745	4.90E-38
Myc	4.54E-181	-0.338188952	1.36E-177
Paics	2.93E-133	-0.338277838	8.79E-130
Ccl12	4.63E-26	-0.339034353	1.39E-22
Gpr183	2.60E-25	-0.33993052	7.81E-22
Camk1	5.25E-76	-0.340364634	1.57E-72
Cxcl3	1.68E-13	-0.341270427	5.03E-10
Hint1	1.74E-36	-0.341816653	5.23E-33
Abhd5	3.97E-209	-0.342628848	1.19E-205
Ddost	6.39E-52	-0.343215058	1.92E-48
Ndufc1	2.13E-46	-0.344295619	6.38E-43
Ccl17	5.26E-43	-0.345011689	1.58E-39
Aplp2	7.00E-76	-0.345846866	2.10E-72
B3gnt8	6.48E-83	-0.348026175	1.94E-79
Dbi	1.20E-34	-0.353058339	3.61E-31
Plaur	2.06E-20	-0.353279383	6.19E-17
Pnp	3.83E-61	-0.357291927	1.15E-57
Slc40a1	8.40E-07	-0.357350169	0.00251896
Casp6	3.16E-123	-0.359011274	9.47E-120
Tmem176a	1.08E-18	-0.360041895	3.23E-15
Odc1	4.85E-147	-0.36012329	1.45E-143
Csf2rb	4.67E-41	-0.360223792	1.40E-37
Cpne2	9.56E-56	-0.3609384	2.87E-52
Bst1	1.83E-09	-0.361777147	5.49E-06
Tubb5	1.48E-26	-0.362264014	4.45E-23
Chd9	2.25E-32	-0.363205218	6.74E-29
Cysltr1	1.71E-30	-0.363864962	5.14E-27
Itgam	2.10E-39	-0.364588476	6.31E-36
Tgm2	8.37E-10	-0.364871894	2.51E-06
Rps27l	1.84E-36	-0.365220809	5.52E-33
Adssl1	7.66E-30	-0.367421134	2.30E-26
Atp13a2	1.34E-36	-0.368728503	4.02E-33
Rrbp1	3.44E-43	-0.37128454	1.03E-39
Prdx4	5.31E-96	-0.375553074	1.59E-92
Klc4	6.97E-268	-0.380437529	2.09E-264
Ptafr	8.17E-58	-0.384554096	2.45E-54
Runx3	1.68E-57	-0.386580073	5.03E-54

Gas7	1.73E-55	-0.388494841	5.19E-52
Mertk	3.01E-71	-0.389693882	9.03E-68
Ttc39c	1.86E-282	-0.389951048	5.57E-279
Gclm	6.53E-185	-0.390456961	1.96E-181
Got1	2.85E-209	-0.391102764	8.54E-206
Ap3s1	5.95E-78	-0.3920743	1.79E-74
Hist1h2bc	4.65E-17	-0.392311565	1.40E-13
Ncf1	3.53E-59	-0.392404498	1.06E-55
Ctss	2.32E-32	-0.392435302	6.96E-29
Trf	3.57E-25	-0.399025867	1.07E-21
Ifi207	8.66E-64	-0.399478527	2.60E-60
Gatm	2.67E-09	-0.401162409	8.01E-06
Mrpl54	2.43E-62	-0.401980032	7.29E-59
Uck2	5.74E-104	-0.402078815	1.72E-100
Igsf8	4.72E-124	-0.404926805	1.42E-120
Tubb4b	8.61E-98	-0.404929582	2.58E-94
Atf3	1.08E-55	-0.406981863	3.25E-52
Timp1	5.26E-67	-0.407294766	1.58E-63
Itgb5	2.39E-138	-0.407822839	7.18E-135
Tfec	1.40E-86	-0.408553913	4.20E-83
Edem1	3.37E-89	-0.409956935	1.01E-85
Myof	1.28E-74	-0.416304759	3.84E-71
Rab7b	1.04E-80	-0.417641804	3.13E-77
Manf	5.26E-64	-0.41905846	1.58E-60
Mgst1	3.46E-21	-0.421634213	1.04E-17
Ntpcr	2.43E-118	-0.422298872	7.29E-115
Ccdc86	1.47E-90	-0.423278458	4.42E-87
Snx5	1.63E-28	-0.426901363	4.88E-25
Gcsh	1.33E-195	-0.426904998	3.98E-192
Apoc1	9.96E-39	-0.427146546	2.99E-35
Mrc1	1.59E-75	-0.428773985	4.77E-72
Tmem176b	1.24E-05	-0.429872267	0.037123508
C1qbp	1.78E-82	-0.434261285	5.34E-79
Capn2	1.52E-91	-0.434621494	4.55E-88
Ranbp1	5.30E-65	-0.437825975	1.59E-61
Slc3a2	1.19E-64	-0.439947533	3.56E-61
Ms4a6c	5.33E-32	-0.441629494	1.60E-28
Tbxas1	2.10E-188	-0.442614254	6.30E-185

Scpep1	4.30E-49	-0.442619357	1.29E-45
Xbp1	7.70E-42	-0.442945835	2.31E-38
Rflnb	4.28E-59	-0.444112921	1.28E-55
Pycard	1.42E-45	-0.448439646	4.26E-42
Rgcc	3.93E-72	-0.450362089	1.18E-68
Gde1	8.10E-97	-0.450865634	2.43E-93
Blvra	1.66E-114	-0.453611734	4.98E-111
Lat2	5.99E-85	-0.454087527	1.80E-81
Ap1s1	4.66E-154	-0.455452728	1.40E-150
Ppia	3.71E-79	-0.455862356	1.11E-75
F10	8.63E-22	-0.462211127	2.59E-18
Lrp1	1.39E-41	-0.46833155	4.16E-38
Hmgn2	7.66E-74	-0.47090135	2.30E-70
Cxcl14	2.49E-44	-0.476162357	7.48E-41
Rab3il1	2.49E-119	-0.476798844	7.48E-116
Krtcap2	2.07E-73	-0.476935048	6.20E-70
Hsp90b1	2.28E-51	-0.480348853	6.85E-48
Tubb2a	8.73E-113	-0.481078621	2.62E-109
Pira2	1.39E-129	-0.482465522	4.17E-126
Dusp22	1.14E-110	-0.4837559	3.42E-107
Aldh2	6.56E-54	-0.487560626	1.97E-50
Cd177	1.50E-14	-0.48894519	4.50E-11
Pkib	1.26E-18	-0.490572354	3.79E-15
Gm46224	5.92E-228	-0.494103091	1.78E-224
Jun	2.79E-27	-0.497325665	8.38E-24
Hexa	4.07E-54	-0.49838116	1.22E-50
Cttnbp2nl	6.80E-110	-0.499042793	2.04E-106
C5ar1	4.73E-44	-0.499138561	1.42E-40
Ptms	7.60E-67	-0.50047455	2.28E-63
Slc7a11	6.14E-69	-0.501353569	1.84E-65
Adam15	1.09E-87	-0.501662442	3.26E-84
Mcub	8.38E-94	-0.504313768	2.51E-90
Socs3	4.90E-32	-0.505807628	1.47E-28
S100a4	7.39E-28	-0.505998785	2.22E-24
Sh3pxd2b	2.53E-258	-0.509662274	7.59E-255
Cmklr1	1.62E-213	-0.512319966	4.87E-210
Clec4d	1.31E-50	-0.516367984	3.93E-47
Mmp9	1.77E-40	-0.51718551	5.30E-37

Rbpj	1.80E-240	-0.517394984	5.40E-237
Ssr4	8.38E-63	-0.519141794	2.51E-59
Vat1	1.70E-81	-0.521228549	5.11E-78
Plekho1	3.94E-89	-0.521945385	1.18E-85
Aif1	1.70E-105	-0.523984382	5.11E-102
Ms4a6b	2.11E-52	-0.533567966	6.33E-49
P2rx4	2.52E-104	-0.534848948	7.55E-101
Fam129b	1.42E-124	-0.5359534	4.25E-121
Tmem37	3.62E-67	-0.536176602	1.09E-63
Sdf2l1	5.02E-163	-0.542985787	1.51E-159
Impdh2	1.00E-137	-0.544318672	3.01E-134
Hif1a	9.77E-163	-0.547212196	2.93E-159
Mt2	9.23E-76	-0.552154341	2.77E-72
Spint1	8.63E-109	-0.558727311	2.59E-105
H2afz	3.91E-51	-0.567435263	1.17E-47
Anxa4	7.47E-103	-0.577028055	2.24E-99
Atp5g1	5.25E-71	-0.579167896	1.57E-67
Hmgb1	9.19E-72	-0.579234621	2.76E-68
Fkbp1a	7.84E-70	-0.58227958	2.35E-66
Hmgn1	2.00E-266	-0.584793649	5.99E-263
Grina	6.55E-58	-0.588018655	1.96E-54
Tpi1	7.98E-52	-0.591333037	2.39E-48
Hspa5	7.36E-38	-0.592332434	2.21E-34
Ms4a4a	4.85E-35	-0.592729788	1.46E-31
Dok2	4.67E-57	-0.593873527	1.40E-53
Mmp14	7.37E-37	-0.593912784	2.21E-33
Lilrb4a	4.48E-74	-0.595527166	1.34E-70
Psap	1.06E-60	-0.598768812	3.18E-57
Ms4a6d	1.18E-82	-0.59887385	3.54E-79
Gyg	8.97E-59	-0.601085968	2.69E-55
Maf	2.04E-114	-0.602612125	6.12E-111
Hexb	8.66E-78	-0.606482858	2.60E-74
Bnip3	3.03E-63	-0.609822376	9.08E-60
Ifitm1	1.57E-27	-0.614063782	4.71E-24
Gm14005	8.02E-109	-0.614510634	2.41E-105
Syngr2	7.03E-74	-0.615215869	2.11E-70
P4hb	8.82E-78	-0.617805819	2.64E-74
Rnase4	1.17E-165	-0.627389328	3.52E-162

Calr	3.63E-75	-0.628922176	1.09E-71
Comt	1.24E-141	-0.629010132	3.72E-138
Tmem86a	5.04E-62	-0.63226338	1.51E-58
Stfa2l1	3.38E-07	-0.633081153	0.001013986
Gm26917	3.15E-66	-0.645300372	9.44E-63
Il1rn	6.47E-18	-0.654615114	1.94E-14
S100a1	7.51E-193	-0.663363182	2.25E-189
Pdia6	2.42E-111	-0.664630801	7.25E-108
Pa2g4	8.78E-164	-0.665890281	2.63E-160
Tagln2	2.02E-60	-0.677057813	6.07E-57
Pld3	1.73E-189	-0.684629978	5.20E-186
Prdx5	3.86E-70	-0.684785183	1.16E-66
Stab1	5.84E-86	-0.686224513	1.75E-82
P2ry6	1.14E-103	-0.688823971	3.42E-100
Alas1	3.57E-119	-0.710004831	1.07E-115
2010111I01Rik	5.01E-173	-0.711063412	1.50E-169
Ccl9	8.27E-71	-0.712237296	2.48E-67
BC100530	3.23E-39	-0.715030533	9.69E-36
Anxa5	6.63E-88	-0.719537068	1.99E-84
H2-DMa	1.57E-72	-0.726431957	4.70E-69
Hopx	1.05E-145	-0.727103805	3.16E-142
Nme2	9.94E-126	-0.736022122	2.98E-122
Cd93	2.94E-60	-0.7427382	8.82E-57
Ahnak	2.28E-65	-0.742812991	6.84E-62
Lamp1	3.23E-130	-0.743687836	9.70E-127
Atpif1	3.02E-86	-0.753766148	9.07E-83
Emp1	6.48E-125	-0.756604031	1.94E-121
Camp	3.39E-140	-0.762256244	1.02E-136
Ssr2	3.20E-138	-0.778777443	9.59E-135
Clec5a	2.87E-62	-0.781359924	8.61E-59
Apoe	1.34E-25	-0.784795802	4.03E-22
Ftl1-ps1	7.07E-51	-0.789523759	2.12E-47
Cd24a	7.65E-78	-0.791774542	2.30E-74
Clec4a2	5.13E-106	-0.794243907	1.54E-102
Ms4a7	5.09E-15	-0.800065675	1.53E-11
Prdx1	4.49E-46	-0.804096519	1.35E-42
Tuba1c	2.00E-87	-0.804229052	6.00E-84
Nhp2	6.44E-179	-0.810370544	1.93E-175

Anxa1	1.48E-79	-0.831731082	4.43E-76
H2-DMb1	4.51E-29	-0.836366683	1.35E-25
Ccl7	1.58E-93	-0.838258128	4.73E-90
Dab2	1.26E-131	-0.841877285	3.79E-128
Cd68	5.84E-78	-0.844912823	1.75E-74
Esd	2.45E-83	-0.846494695	7.34E-80
Fcgr1	7.03E-111	-0.85040657	2.11E-107
Rgs10	5.18E-89	-0.853016501	1.55E-85
Cndp2	1.64E-130	-0.855307285	4.91E-127
Cd38	7.03E-170	-0.855587226	2.11E-166
Glrx	7.08E-116	-0.856734892	2.13E-112
Cfp	1.36E-72	-0.864015188	4.09E-69
Ly6c2	4.65E-110	-0.869082904	1.39E-106
Fcgr2b	6.24E-87	-0.893486396	1.87E-83
Aldoa	2.49E-111	-0.910445352	7.46E-108
Ly86	1.77E-111	-0.910796409	5.32E-108
Lrg1	1.74E-43	-0.914437515	5.21E-40
Cd33	7.66E-201	-0.919583308	2.30E-197
Tmem256	4.70E-140	-0.920668596	1.41E-136
Lmna	4.32E-124	-0.921173111	1.30E-120
Slc25a4	1.27E-174	-0.921804565	3.80E-171
Ninj1	2.06E-87	-0.92857693	6.17E-84
Gpnmb	1.14E-51	-0.935466955	3.41E-48
Clec4n	3.03E-104	-0.954062903	9.08E-101
Vcan	2.37E-08	-0.957419425	7.11E-05
Crip1	4.45E-74	-0.963442812	1.34E-70
Anxa3	6.46E-232	-0.965598292	1.94E-228
Hsp90ab1	4.35E-153	-0.968103362	1.31E-149
Ctsz	4.32E-115	-0.97012941	1.30E-111
Cxcl2	5.44E-92	-0.977353454	1.63E-88
Tuba1b	3.85E-135	-0.978448082	1.16E-131
Pgam1	3.09E-94	-0.981221962	9.27E-91
Aprt	7.23E-125	-0.989165108	2.17E-121
Slpi	1.10E-36	-0.989400284	3.30E-33
Adam8	2.47E-74	-0.995522569	7.41E-71
Pgk1	4.26E-116	-1.00118855	1.28E-112
Akr1a1	1.71E-153	-1.007367381	5.12E-150
Msr1	2.71E-200	-1.068478994	8.13E-197

Ccl5	2.25E-217	-1.071490185	6.76E-214
Sdc4	4.32E-160	-1.080135713	1.30E-156
Mmp8	1.54E-55	-1.100950079	4.61E-52
Lgals3	1.52E-158	-1.108478119	4.55E-155
Anxa2	1.47E-119	-1.128975385	4.41E-116
Prdx6	7.74E-166	-1.129066171	2.32E-162
Ctsd	1.05E-107	-1.150513224	3.15E-104
Prtn3	7.75E-29	-1.151136018	2.32E-25
Cd14	1.34E-122	-1.171451299	4.01E-119
Grn	3.69E-147	-1.175539098	1.11E-143
Hmox1	6.68E-58	-1.192884136	2.00E-54
Tmsb10	8.67E-137	-1.20375486	2.60E-133
Mif	1.04E-122	-1.204157525	3.13E-119
Lilr4b	2.21E-152	-1.221765558	6.62E-149
Ctsh	6.40E-185	-1.226605634	1.92E-181
App	9.02E-175	-1.236170008	2.71E-171
Lyz2	3.27E-170	-1.237630756	9.81E-167
Mmp12	1.38E-94	-1.241314701	4.13E-91
C3ar1	4.00E-181	-1.272709147	1.20E-177
Cxcl16	8.34E-255	-1.28224628	2.50E-251
Npc2	4.50E-236	-1.298545754	1.35E-232
Selenop	2.17E-55	-1.342111922	6.52E-52
Fth1	4.22E-111	-1.347878764	1.27E-107
Chil3	2.66E-15	-1.471641532	7.98E-12
Fcgr3	4.34E-202	-1.484530623	1.30E-198
S100a6	1.03E-185	-1.492281874	3.09E-182
Fabp5	1.88E-101	-1.530112213	5.64E-98
Atp6v0c	1.31E-226	-1.565992405	3.92E-223
Lgmn	1.07E-139	-1.620937587	3.22E-136
Cd63	8.89E-111	-1.622827274	2.67E-107
Pf4	2.06E-44	-1.625382608	6.18E-41
Trem2	9.76E-275	-1.649569611	2.93E-271
Il1r2	1.83E-144	-1.660269903	5.50E-141
Ccr1	6.53E-271	-1.690532312	1.96E-267
AA467197	4.60E-63	-1.725938652	1.38E-59
Ctsc	4.69E-193	-1.727399674	1.41E-189
Cstb	8.11E-164	-1.780398001	2.43E-160
Ngp	5.01E-198	-1.801517221	1.50E-194

Lgals1	2.48E-168	-1.83505833	7.44E-165
Tgfbi	1.54E-248	-1.859701525	4.62E-245
S100a10	1.28E-220	-1.871578196	3.83E-217
F13a1	1.20E-130	-1.880324102	3.61E-127
Ier3	4.87E-181	-1.883158192	1.46E-177
Capg	3.11E-232	-1.901042407	9.34E-229
Ccl2	4.89E-63	-1.923117525	1.47E-59
Ecm1	7.81E-259	-1.98305014	2.34E-255
Ccr2	5.41E-181	-1.994442495	1.62E-177
Wfdc21	8.35E-116	-1.996398564	2.51E-112
Mafb	2.69E-213	-2.007547594	8.06E-210
S100a9	9.99E-11	-2.056404129	3.00E-07
H2-Eb1	3.00E-08	-2.070637609	9.01E-05
Lcn2	7.54E-116	-2.103288558	2.26E-112
H2-Ab1	2.28E-09	-2.190320969	6.84E-06
Ctsl	1.58E-269	-2.216567253	4.73E-266
Wfdc17	1.51E-235	-2.229468315	4.53E-232
S100a8	9.70E-102	-2.264810493	2.91E-98
Thbs1	1.50E-179	-2.414328695	4.50E-176
H2-Aa	5.73E-129	-2.45098969	1.72E-125
Mt1	1.85E-275	-2.51755963	5.56E-272
C1qa	1.49E-55	-2.553358363	4.48E-52
Cd74	8.15E-51	-2.641931942	2.45E-47
Arg1	2.20E-129	-2.753891471	6.59E-126
Vim	7.62E-259	-2.974375345	2.28E-255
C1qc	7.32E-106	-2.974413031	2.19E-102
Spp1	2.53E-52	-3.348673888	7.60E-49
C1qb	6.86E-87	-3.356198186	2.06E-83
Fn1	1.28E-287	-3.470380179	3.83E-284
